# Understanding visual impairment trends in the Gulf Council Countries: An analysis from 1990 to 2021 and time-series predictions for 2030

**DOI:** 10.1371/journal.pone.0357032

**Published:** 2026-09-08

**Authors:** Ahmed Saad AL Zomia, Saeed T. Alshahrani, Ibrahim Ali AL Zehefa, Abdullah Jallwi Korkoman, Ahmed Abdullah Alamoud, Mazen Abdullah Alqahtani, Mahmoud Tarek Mirdad, Mohammed Tarek Mirdad, Raad Ahmed A. Alnami, Faisal Nasser M. Alahmari, Saad Ali Alshahrani, Sultan Abdulrahman Alyali

**Affiliations:** 1 Asir Central Hospital, Abha, Saudi Arabia; 2 Ophthalmology Department, King Fahad Medical City, Riyadh, Saudi Arabia; 3 Faculty of Medicine, King Khalid University, Abha, Saudi Arabia; 4 Department of Ophthalmology, Prince Sultan Military Medical City, Riyadh, Saudi Arabia; 5 Saudi German Hospital, Abha, Saudi Arabia; 6 Pediatric Ophthalmology Department, KKESH, Riyadh, Saudi Arabia; 7 Muhayil Health Sector, Muhayil General Hospital, Aseer Health Cluster, Abha, Saudi Arabia; 8 Ophthalmology Department, Aseer Central Hospital, Abha, Saudi Arabia; 9 Riyadh First Health Cluster, Riyadh, Saudi Arabia; University of Pittsburgh, UNITED STATES OF AMERICA

## Abstract

**Background:**

This research investigates blindness prevalence trends in Gulf Cooperation Council (GCC) countries from 1990 to 2021 and provides projections up to 2030. The study aimed to inform public health planning, policy formulation, and healthcare delivery in the region.

**Methods:**

Using data from the Global Burden of Disease Study 2021, we conducted a time-series analysis applying AutoRegressive Integrated Moving Average (ARIMA) models to evaluate age-standardized prevalence rates and Disability-Adjusted Life Years (DALYs) for blindness. Country-specific and gender-specific were analyzed. Forecasts for 2022–2030 were generated and validated against observed data.

**Results:**

Between 1990 and 2021, most GCC countries exhibited declining trends in blindness prevalence. Bahrain’s rate decreased from 13,498.2 to 12,884.7 per 100,000, Kuwait from 12,873.8 to 12,881.4, Saudi Arabia from 14,207.2 to 14,166.8, and the UAE from 11,905.9 to 11,869.6. Oman and Qatar showed relative stability with minor fluctuations. Gender disparities were consistent, with higher prevalence among females in all countries except the UAE. DALYs also declined across the region, with Saudi Arabia reporting the highest burden. Forecasts for 2022–2030 indicate continued reductions in most countries, though Oman may experience a moderate increase.

**Conclusions:**

The findings underscore the need for targeted public health strategies, improved healthcare infrastructure, and gender-sensitive interventions to address visual impairment in the GCC. Continuous monitoring and international collaboration are essential to sustain progress and mitigate future burdens.

## Introduction

In many countries across the world, the average age of the population is rising, more people are reaching adulthood due to rising sociodemographic status and life expectancy, and the burden of disease is shifting toward non-communicable diseases (NCDs) and disabilities. This epidemiological transition affects the majority of the primary causes of vision impairment, such as cataracts and undercorrected refractive error [1], and they come at a large financial and societal cost to each individual. [2,3]

According to the latest global report on blindness and vision impairment among individuals aged 50 and above, approximately 43.3 million people worldwide are blind, and 295 million people suffer from moderate to severe vision impairment as of 2020. [4] Blindness and vision loss were expected to have caused 22.6 million disability-adjusted life years (DALYs) globally in 2019, making up 0.88% of all DALYs caused by all causes. [5] Severe personal financial and educational challenges are brought on by blindness and vision loss, [6] which also lowers the quality of life [7] and raises mortality. It is possible that about one-third of the patients suffering from visual impairment did not obtain appropriate preventive and therapeutic care. [8]

Saudi Arabia, Kuwait, the United Arab Emirates (UAE), Qatar, Bahrain, and Oman are the six Middle Eastern countries that make up the Gulf Cooperation Council (GCC), a political and economic partnership. In May 1981, the GCC was founded in Riyadh, Saudi Arabia. The GCC aims to unite its members via shared goals and comparable political and cultural identities founded on Islamic principles. [9] The combined land area of all member states is 2.57 million square kilometers, with a total population of approximately 58.86 million people.[10]

To reduce the growing burden of vision impairment, GCC member states have adopted strong national health strategies and regional collaborative frameworks [11]. Since the 1990s, countries such as Oman and Saudi Arabia have pioneered integrated primary eye care (PEC) models, in which vision screening is routinely included in primary healthcare services [12]. At the regional level, the Prevention of Blindness Union (PBU) and the Middle East Africa Council of Ophthalmology (MEACO)—often under Saudi leadership—have played a key role in coordinating “Vision 2020: The Right to Sight” initiatives across the Gulf. In addition, the healthcare reform objectives of Saudi Vision 2030 and the UAE National Agenda have increasingly prioritized preventive care, with particular attention to the early detection of diabetic retinopathy and glaucoma, both of which are highly prevalent in the region due to rising metabolic risk factors [11,13,14]. These efforts are further reinforced by the Gulf Cooperation Council Forum on Avoidable Blindness, which provides a platform for harmonizing clinical guidelines and allocating resources to meet the specific epidemiological needs of Gulf populations [15].

The population over 50 has been analyzed for the updated worldwide and regional prevalence of blindness and vision loss, which is classified by the severity of visual impairment, in addition to eye diseases, which provides comprehensive knowledge for understanding the landscape of blindness and vision loss burden. [4,16] However, the impact of putative risk factors and the burden on the entire population have not yet been evaluated. Using a wide range of data sources and reliable statistical techniques, the most recent Global Burden of Disease (GBD) 2019 Study calculated the annual burden of 369 diseases for 204 nations and territories between 1990 and 2019, which provided a unique opportunity to grasp the progress of the blindness and vision loss burden. [5]

Our study hypothesis aimed at comprehensively understanding visual impairment trends in the GCC from 1990 to 2021 and forecasting for 2022–2030. We hypothesized that there has been a significant temporal change in the prevalence of blindness and vision loss within the GCC over the specified period. This study aimed to fill critical gaps in our understanding of blindness and vision loss trends within the GCC, focusing on the period from 1990 to 2021 and providing predictions for 2022–2030 through robust time-series analysis. By leveraging data from the GBD 2021 study, our investigation assessed the evolving burden of visual impairment and its associated risk factors.

## Methodology

### Study design and data sources

This retrospective time-series analysis utilized secondary data from the Global Burden of Disease Study 2021 (GBD 2021), the most recent comprehensive dataset publicly available from the Institute for Health Metrics and Evaluation (IHME). The study encompassed all six Gulf Cooperation Council (GCC) countries: Saudi Arabia, United Arab Emirates, Kuwait, Qatar, Bahrain, and Oman. We analyzed data from 1990 to 2021 for visual impairment metrics, including prevalence rates and Disability-Adjusted Life Years (DALYs), with age-standardized values for all age groups and both sexes.

### Data extraction and Management

Data extraction followed a systematic protocol using the GBD Results Tool and the Global Health Data Exchange (GHDx) platform. We extracted:

- Age-standardized prevalence rates per 100,000 population
- DALY rates (sum of Years of Life Lost [YLL] and Years Lived with Disability [YLD])
- Uncertainty intervals (upper and lower bounds) for all estimates

Initial data compilation and organization were performed in Microsoft Excel 365 to ensure structural consistency across countries and years. The dataset was structured with columns for location, year, measure, metric, value, and uncertainty intervals.

### Data preprocessing and quality control

Comprehensive data cleaning and validation procedures were implemented in R version 4.3.2:

1. Missing Data Handling: No missing values were encountered in the core dataset as GBD provides complete time-series estimates through sophisticated modeling techniques.
2. Outlier Detection: We employed Tukey’s method using the IQR criterion, identifying potential outliers as values beyond Q1 - 1.5 × IQR or Q3 + 1.5 × IQR. However, given the modeled nature of GBD estimates, no data points were excluded as all values represented plausible epidemiological estimates.
3. Data Transformation: Variables were assessed for normality and stationarity requirements. Logarithmic transformations were applied where necessary to stabilize variance, and differencing was used to achieve stationarity for time-series modeling.
4. Consistency Validation: Cross-validation with previous GBD publications ensured data consistency and reliability.

### Statistical analysis framework

#### Descriptive and comparative analysis.

We conducted comprehensive descriptive analyses including:

- Temporal trend analysis with annual percentage changes
- Data visualization through line graphs, and comparative bar charts
- Summary statistics including means, medians, ranges, and interquartile ranges

### Time-series modeling and forecasting

The analytical approach followed the Box-Jenkins methodology for ARIMA modeling:

1. Model Assumptions and Diagnostics: The ARIMA forecasting framework was governed by eight foundational assumptions to ensure the validity and reliability of the 2022–2030 projections. First, the stationarity of each country-specific time series was achieved through appropriate differencing (d) and confirmed via Augmented Dickey-Fuller (ADF) and KPSS tests (p < 0.05 post-differencing). The model assumed linearity, implying that future values maintain a linear relationship with historical data, and parameter constancy, where model coefficients were assumed to remain stable over the forecast horizon. To validate the model’s fit, we confirmed error independence through Ljung-Box tests (p > 0.05 for all series), ensuring residuals were free of autocorrelation. Furthermore, the normality of residuals was verified using Shapiro-Wilk tests and Q-Q plots, while homoscedasticity was confirmed through visual inspection of residual plots, which exhibited no systematic patterns of variance. Finally, the projections operated under the assumptions of data quality accepting GBD 2021 estimates as the baseline and the absence of catastrophic external shocks (e.g., future pandemics or economic crises) that could fundamentally alter the established epidemiological trajectories.
2. Model Identification:
  - Autocorrelation Function (ACF) plots identified Moving Average (MA) components (q)
  - Partial Autocorrelation Function (PACF) plots identified AutoRegressive (AR) components (p)
  - Automatic differentiation via ndiffs() function determined integration order (d)
3. Model Selection Criteria:
  - Akaike Information Criterion (AIC) for model fit comparison
  - Bayesian Information Criterion (BIC) for parsimony preference
  - Minimum MAPE (Mean Absolute Percentage Error) for forecast accuracy
4. Model Estimation and Validation:
  - Maximum likelihood estimation of ARIMA parameters
  - Ljung-Box tests for residual autocorrelation (p > 0.05 indicating adequacy)
  - Residual analysis for normality, homoscedasticity, and independence
  - Key performance metrics for assessing model accuracy include MAE (Mean Absolute Error), which indicates the average size of errors for clearer understanding; RMSE (Root Mean Squared Error), which emphasizes larger errors by squaring them before averaging, making it more responsive to outliers; and MAPE (Mean Absolute Percentage Error), which presents the error as a percentage of the actual values, providing a scale-independent assessment of relative accuracy.
5. Forecast Generation:
  - Short-term projections: 2022–2024 for immediate policy applications
  - Long-term projections: 2025–2030 for strategic health planning
  - 95% prediction intervals to quantify forecast uncertainty

### Sensitivity analysis

To assess the robustness and stability of our primary ARIMA forecasting models, we conducted a comprehensive multi-faceted sensitivity analysis. This approach aimed to evaluate how variations in model specifications, training data periods, and alternative methodological frameworks could influence our projections for blindness prevalence.

First, a parameter sensitivity analysis was performed by testing a range of adjacent ARIMA $(p,d,q)$ specifications around the optimal model identified by the auto.arima function. This allowed us to evaluate the stability of parameter estimates and forecast consistency across plausible alternative model orders.

Second, a training window sensitivity analysis was conducted by sequentially refitting the models using different historical time horizons (e.g., 1990–2010 vs. 1990–2021). This approach assessed the influence of more recent observations on forecast accuracy and evaluated whether the underlying temporal trends were stable over time.

Third, we executed a rigorous benchmarking exercise to compare the relative forecasting performance of our primary ARIMA models against a suite of well-established statistical baselines. This included Simple Exponential Smoothing (SES) and the broader Exponential Smoothing framework (ETS), which captures Error, Trend, and Seasonality components. Forecast accuracy metrics—including Mean Absolute Error (MAE), Root Mean Squared Error (RMSE) and Mean Absolute Percentage Error (MAPE) were calculated for each competing model to validate the superiority and reliability of our chosen approach. **Supplementary tables**

### Joinpoint regression analysis

To identify significant temporal breakpoints in blindness prevalence trends, we conducted joinpoint regression analysis using the segmented package in R version 4.3.2. This method fits log-linear segmented regression models to the time-series data, allowing detection of statistically significant joinpoints where trends change direction or magnitude. We tested for 0, 1, and up to 2 joinpoints over the study period (1990−2021), with the optimal number selected using permutation tests (α = 0.05) with Bonferroni correction. For each identified segment, the Annual Percentage Change (APC) was calculated as APC = (e^β – 1) × 100, where β represents the segment-specific slope coefficient. Minimum segment length was restricted to three years to ensure model stability.

### Software and reproducibility

All analyses were conducted in R version 4.3.2 using the following packages:

- forecast for arima modeling and forecasting
- tseries for stationarity testing
- ggplot2 for advanced visualization
- dplyr and tidyr for data manipulation
- readxl for data import

Complete reproducible code and data processing scripts have been archived to ensure transparency and facilitate replication studies.

### Ethical considerations

This study utilized exclusively de-identified, aggregate, publicly available data from the GBD study. No ethical approval was required as the research involved secondary analysis of anonymized population-level data. [17]

## Results

### Prevalence of blindness and vision loss (Fig 1)

Between 1990 and 2021, Bahrain experienced a gradual decline in the age-standardized prevalence of blindness and vision loss. In 1990, the overall prevalence was 13,498.2 cases per 100,000 population, which decreased to 12,884.7 by 2021—a reduction of approximately 4.5%. This downward trend was consistent across both males and females, with male prevalence dropping from 12,186.3 to 11,742.2 and female prevalence declining from 15,289.9 to 14,749.0 over the same period, representing a similar relative decrease of around 3.5% for each group. Notably, females consistently exhibited higher rates of vision loss compared to males throughout the entire timeframe. The most significant reductions occurred between 1990 and 2017, after which the rates began to stabilize, with only minor fluctuations in the final years, including a slight plateau between 2020 and 2021. Kuwait experienced a slight overall decline in the age-standardized prevalence of blindness and vision loss. In 2019, the total prevalence was 12,873.8 cases per 100,000 population, which decreased to 12,881.4 by 2021—a marginal increase of approximately 0.06%. This minimal change reflects a period of relative stabilization after a more pronounced downward trend observed in previous years. Among males, the rate declined from 11,568.8 in 2019–11,539.6 in 2021, representing a reduction of about 0.25%. For females, the prevalence decreased slightly from 14,617.8 in 2019–14,595.1 in 2021, a drop of roughly 0.16%. Throughout this period, females consistently had higher rates of blindness and vision loss compared to males. Oman experienced a slight overall increase in the age-standardized prevalence of blindness and vision loss. In 2019, the total prevalence was 15,152.9 cases per 100,000 population, which rose to 15,143.2 by 2021—a marginal decrease of approximately 0.06%. This minimal change reflects a period of relative stabilization after a more pronounced downward trend observed in previous years. Among males, the rate declined from 14,074.7 in 2019–14,078.9 in 2021, representing a negligible increase of about 0.03%. For females, the prevalence decreased slightly from 16,820.3 in 2019–16,820.7 in 2021, a rise of less than 0.01%. Throughout this period, females consistently exhibited higher rates of vision loss compared to males. Qatar experienced a relatively stable trend in the age-standardized prevalence of blindness and vision loss, with minor fluctuations. In 2019, the overall prevalence was 12,653.5 cases per 100,000 population, which slightly decreased to 12,642.4 in 2020 before rising again to 12,652.9 in 2021—indicating a nearly stable rate with a negligible net decrease of 0.005% over the period. Among males, the prevalence declined from 11,612.7 in 2019–11,586.5 in 2021, a reduction of approximately 0.23%. For females, the rate remained almost unchanged, decreasing slightly from 15,149.7 in 2019–14,979.9 in 2021 (a drop of about 1.1%). Throughout the period, females consistently exhibited higher rates of vision loss compared to males. Saudi Arabia experienced a slight overall decrease in the age-standardized prevalence of blindness and vision loss. In 2019, the total prevalence was 14,207.2 cases per 100,000 population, which decreased to 14,166.8 by 2021—a reduction of approximately 0.28%. This minor decline reflects a period of stabilization after a more pronounced downward trend observed in previous years. Among males, the rate declined from 12,985.7 in 2019–12,964.2 in 2021, representing a drop of about 0.18%. For females, the prevalence decreased slightly from 16,058.9 in 2019–15,994.8 in 2021, a reduction of roughly 0.39%. Throughout this period, females consistently exhibited higher rates of vision loss compared to males. The United Arab Emirates (UAE) experienced a slight overall decline in the age-standardized prevalence of blindness and vision loss. In 2019, the total prevalence was 11,905.9 cases per 100,000 population, which decreased to 11,869.6 by 2021—a reduction of approximately 0.3%. This minor decrease reflects a period of stabilization after a more pronounced downward trend observed in previous years. Among males, the rate declined from 10,786.0 in 2019–10,754.2 in 2021, representing a drop of about 0.3%. For females, the prevalence decreased slightly from 15,380.0 in 2019–15,383.8 in 2021, a negligible increase of less than 0.03%. Throughout this period, females consistently exhibited higher rates of vision loss compared to males.

**Fig 1:**
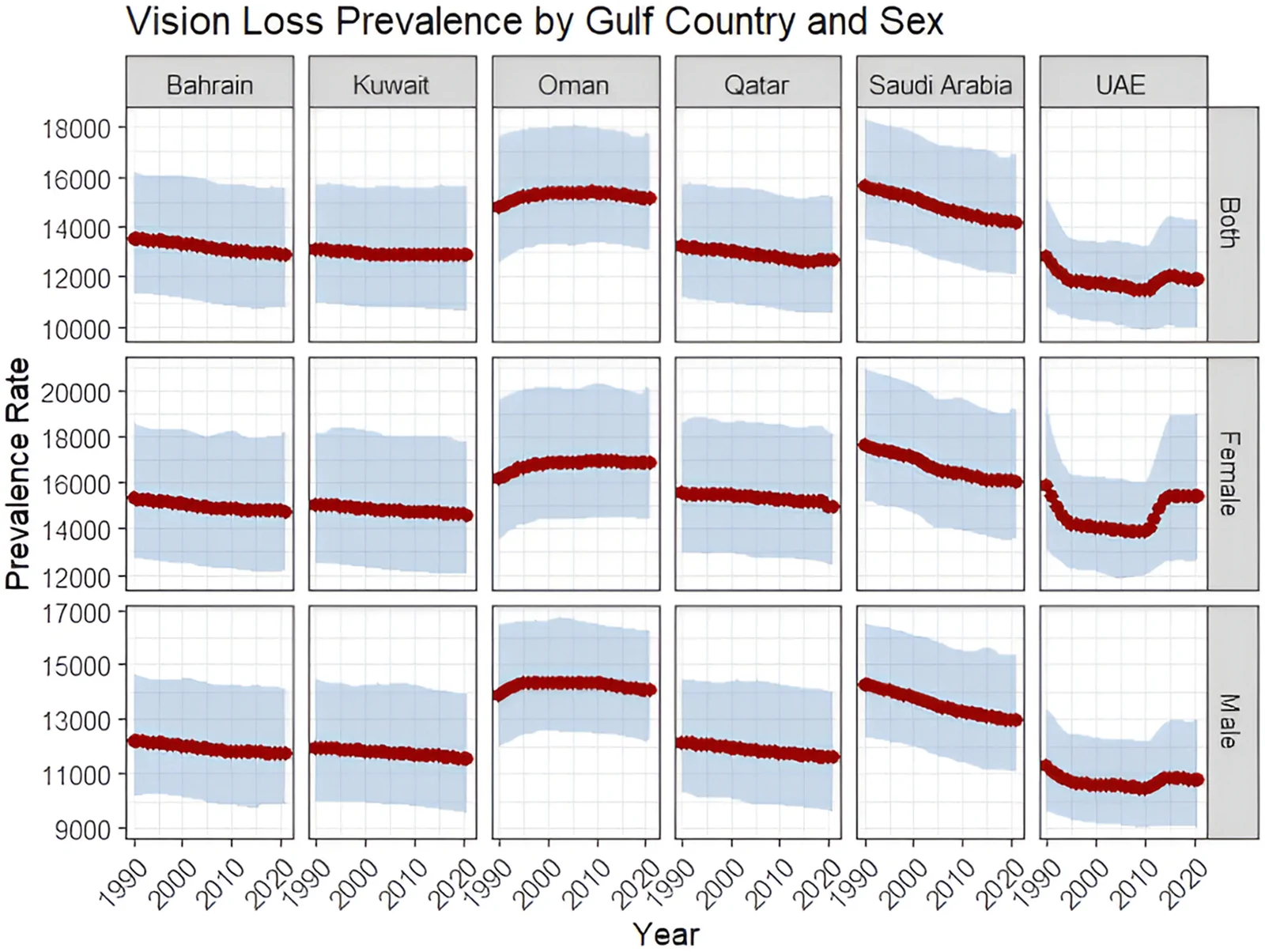
Prevalence of blindness across GCC countries.

### Disability-Adjusted Life Years (DALYs) of blindness and vision loss (Fig 2)

Between 2019 and 2021, Bahrain experienced a consistent decline in the age-standardized burden of blindness and vision loss, as measured by DALYs. In 2019, the total DALY rate was 352.51 per 100,000 population, which decreased to 349.74 by 2021—a reduction of approximately 0.77%. This downward trend was observed across both sexes: the male DALY rate declined from 328.56 in 2019 to 326.83 in 2021, a drop of about 0.53%, while the female rate decreased from 386.16 in 2019 to 382.88 in 2021, a reduction of roughly 0.85%. Throughout this period, females consistently had higher DALY rates than males, reflecting a greater disease burden. Kuwait experienced a slight decline in the age-standardized burden of blindness and vision loss, as measured by Disability-Adjusted Life Years (DALYs). In 2019, the total DALY rate was 336.89 per 100,000 population, which decreased to 336.15 by 2020—a reduction of approximately 0.22%. This minor decline was driven by decreases in both sexes: the male DALY rate fell from 311.76 in 2019 to 310.87 in 2020 (a drop of 0.28%), while the female rate decreased from 371.50 to 370.17 over the same period (a reduction of 0.36%). Throughout this time, females consistently experienced a higher burden of vision loss compared to males. Oman experienced a slight overall decline in the age-standardized burden of blindness and vision loss, as measured by Disability-Adjusted Life Years (DALYs). In 2019, the total DALY rate was 528.73 per 100,000 population, which decreased to 525.88 by 2020—a reduction of approximately 0.54%. This minor decrease was driven by declines in both sexes: the male DALY rate fell from 530.94 in 2019 to 527.77 in 2020 (a drop of 0.60%), while the female rate decreased from 534.49 to 532.32 over the same period (a reduction of 0.41%). Throughout this time, females consistently had a higher burden of vision loss compared to males. Qatar experienced a consistent decline in the age-standardized burden of blindness and vision loss, as measured by Disability-Adjusted Life Years (DALYs). In 2019, the total DALY rate was 351.98 per 100,000 population, which decreased to 348.37 by 2021—a reduction of approximately 1.03%. This downward trend was observed across both sexes: the male DALY rate declined from 321.58 in 2019 to 320.83 in 2021, a drop of about 0.23%, while the female rate decreased from 414.45 in 2019 to 399.98 in 2021, a more substantial reduction of roughly 3.49%. Throughout this period, females consistently had higher DALY rates than males, reflecting a greater disease burden. Saudi Arabia experienced a consistent decline in the age-standardized burden of blindness and vision loss, as measured by Disability-Adjusted Life Years (DALYs). In 2019, the total DALY rate was 535.37 per 100,000 population, which decreased to 526.97 by 2021—a reduction of approximately 1.57%. This downward trend was observed across both sexes: the male DALY rate declined from 500.53 in 2019 to 493.10 in 2021, a drop of about 1.47%, while the female rate decreased from 587.63 in 2019 to 578.27 in 2021, a reduction of roughly 1.59%. Throughout this period, females consistently had higher DALY rates than males, reflecting a greater disease burden. The United Arab Emirates (UAE) experienced a consistent decline in the age-standardized burden of blindness and vision loss, as measured by Disability-Adjusted Life Years (DALYs). In 2019, the total DALY rate was 338.46 per 100,000 population, which decreased to 335.47 by 2021—a reduction of approximately 0.86%. This downward trend was observed across both sexes: the male DALY rate declined from 319.65 in 2019 to 317.21 in 2021, a drop of about 0.76%, while the female rate decreased from 395.26 in 2019 to 392.26 in 2021, a reduction of roughly 0.76%. Throughout this period, females consistently had higher DALY rates than males, reflecting a greater disease burden.

**Fig 2:**
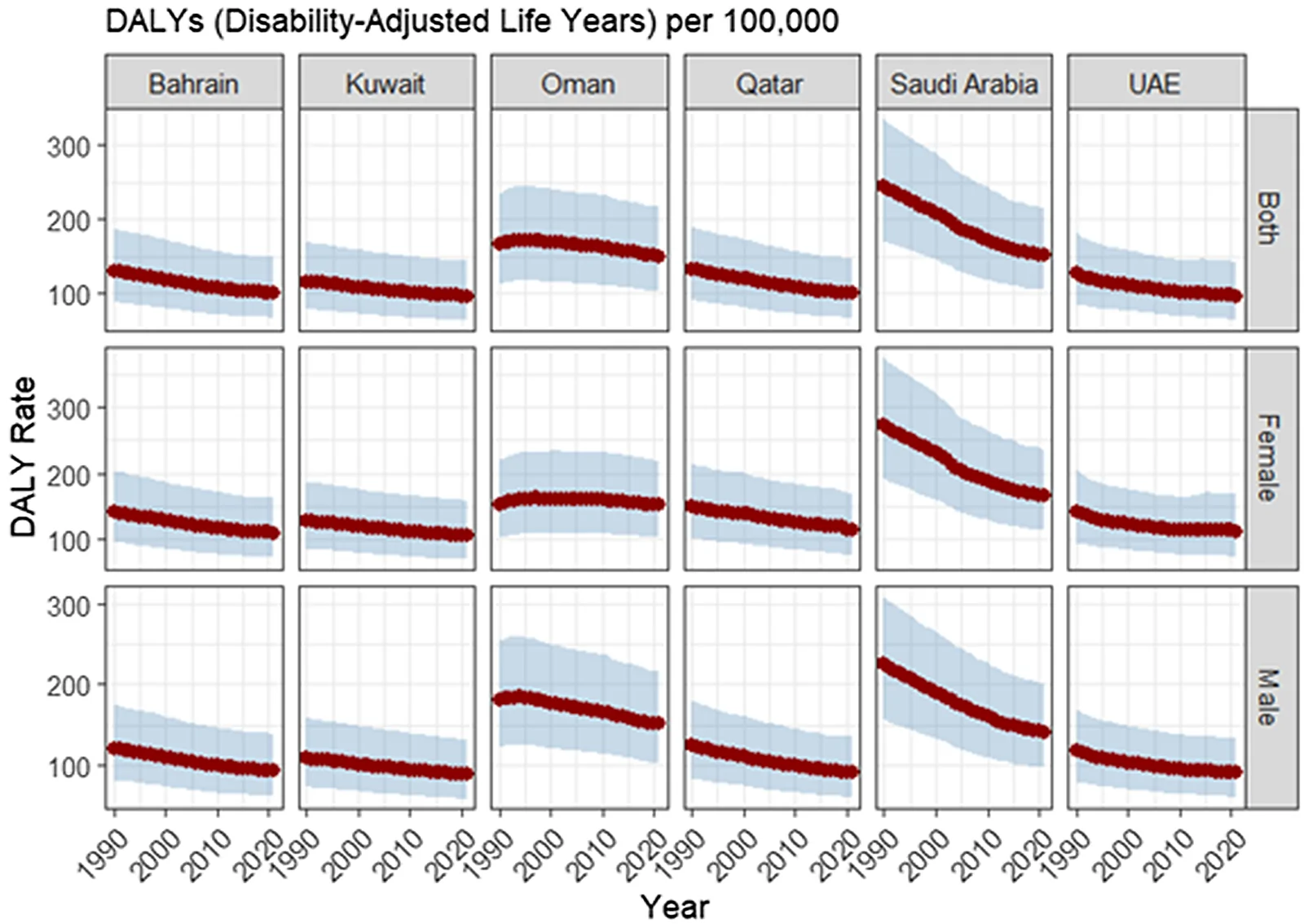
Disability-Adjusted Life Years (DALYs) across GCC countries.

### Forecasted prevalence of blindness in Gulf countries

**Fig 3:**
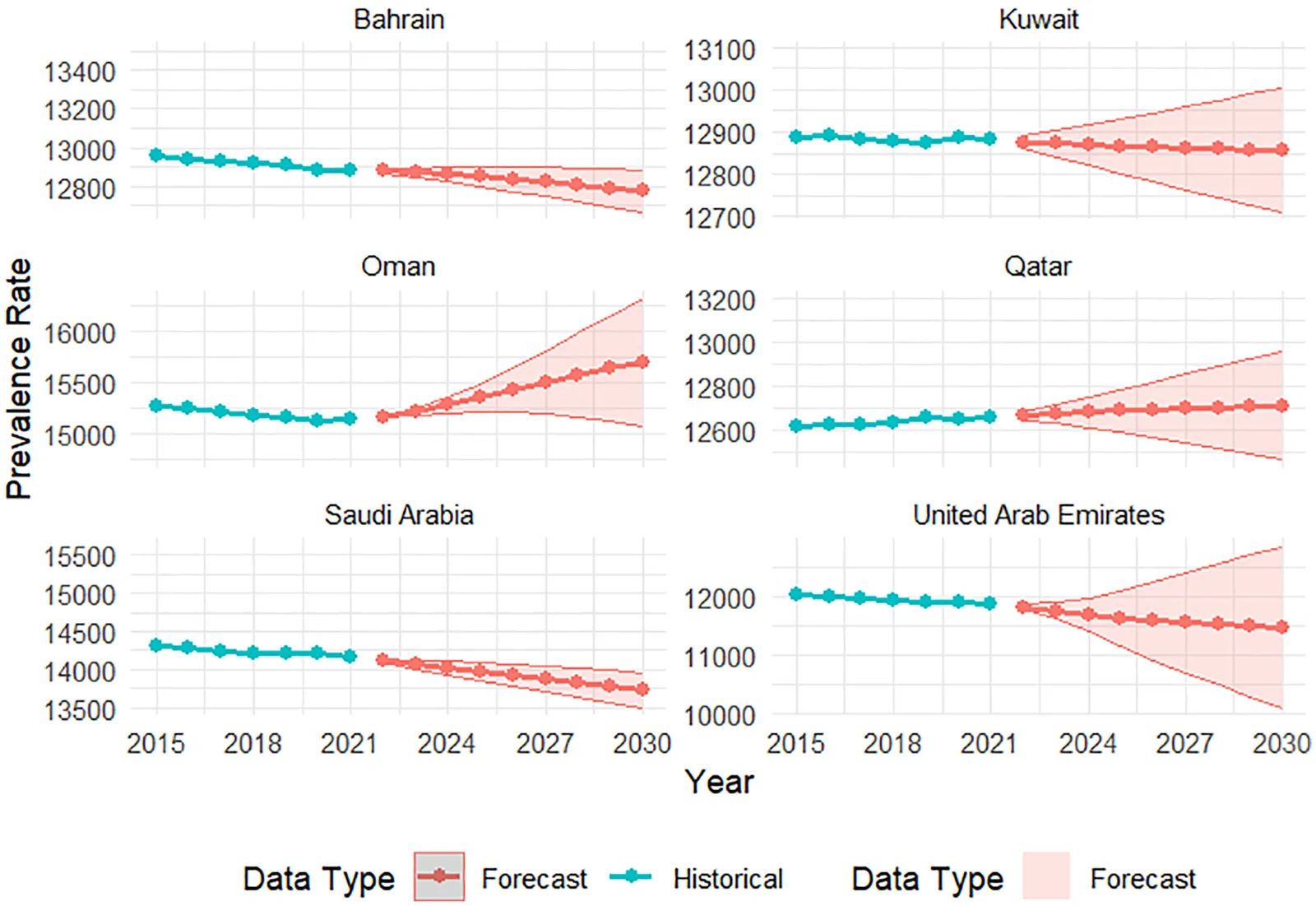
Forecasted prevalence of blindness in gulf countries between 2022–2030.

The forecast of blindness prevalence in Gulf countries for both sexes from 2022 to 2030 shows varying trends across the region. In Bahrain, prevalence is projected to slightly decline, from 12,880 (95% PI: 12,868–12,892) in 2022–12,757 (95% PI: 12,668–12,885) in 2030. Kuwait also shows a mild decline, from 12,876 (95% PI: 12,861–12,891) in 2022–12,858 (95% PI: 12,710–13,005) in 2030, with narrow confidence intervals in the near term, suggesting reliable short-term forecasts. Oman exhibits a clear increasing trend, with prevalence rising from 15,167 (95% PI: 15,154–15,181) in 2022–15,689 (95% PI: 15,066–16,312) in 2030. The widening 95% confidence intervals over time reflect growing uncertainty in longer-term projections. Qatar shows a modest increase, from 12,662 (95% PI: 12,642–12,682) in 2022–12,708 (95% PI: 12,460–12,955) in 2030, indicating relatively stable prevalence with mild upward movement. For Saudi Arabia, prevalence is projected to decrease steadily from 14,115 (95% PI: 14,085–14,146) in 2022–13,727 (95% PI: 13,494–13,959) in 2030, reflecting ongoing improvements in blindness prevention or care. The United Arab Emirates shows a notable decline, from 11,821 (95% PI: 11,778–11,863) in 2022–11,471 (95% PI: 10,110–12,831) in 2030, although the wider confidence intervals toward the end of the period indicate moderate uncertainty in long-term forecasts Fig 3.

The ARIMA models used to forecast blindness prevalence in Gulf countries showed generally good fit for the historical data from 1990 to 2021. The Augmented Dickey-Fuller (ADF) tests indicated that most country-specific time series were stationary after differencing, supporting the use of ARIMA models. The auto.arima() function selected appropriate orders (p,d,q) for each country, capturing the underlying trends and short-term fluctuations in the data. Residual diagnostics, including ACF and PACF of residuals and the Ljung-Box test, suggested no significant autocorrelation remained in most models, indicating that the ARIMA models adequately captured the temporal structure. The narrow 95% confidence intervals in the near-term forecasts (2022–2023) further support the reliability of the models in the short term. However, for longer-term projections (toward 2030), the confidence intervals widen, reflecting increasing uncertainty and the potential influence of unmodeled factors. Overall, the ARIMA models appear suitable for short- to medium-term forecasting of blindness prevalence in Gulf countries, with good statistical fit and appropriate handling of trends and temporal dependencies. Table 1

**Table 1:** Model fit criteria for the predicted forecasted prevalence of blindness in gulf countries between 2022-2030.

Country	ARIMA Order	AIC	BIC	RMSE	MAE	Ljung_Box_p	Stationary Residuals	Forecast Horizon
Bahrain	(1,1,0)	198.5	202.8	5.8	3.7	0.9	Yes	2022-2030
Kuwait	(1,1,0)	213.8	216.6	7.3	5.4	1.0	Yes	2022-2030
Oman	(2,1,2)	217.9	225.1	6.3	4.6	0.8	Yes	2022-2030
Qatar	(1,1,0)	235.1	237.9	9.9	7.8	0.3	Yes	2022-2030
Saudi Arabia	(1,1,0)	261.9	266.2	14.9	9.5	0.5	Yes	2022-2030
United Arab Emirates	(3,1,0)	288.5	294.2	20.3	13.5	0.8	Yes	2022-2030

The forecast of **blindness** DALYs in Gulf countries from 2022 to 2030 shows distinct trends across the region. In Bahrain, DALYs are projected to gradually decrease, from 348.06 (95% CI: 347.57–348.55) in 2022 to 331.79 (95% CI: 320.98–342.60) in 2030, reflecting a steady reduction in disease burden. Similarly, Kuwait shows a decline, from 333.67 (95% CI: 333.09–334.24) in 2022 to 317.64 (95% CI: 308.42–326.86) in 2030, with narrow CIs in the early years, indicating reliable short-term projections. Oman is expected to experience a clear increasing trend in DALYs, rising from 523.74 (95% CI: 522.53–524.95) in 2022 to 537.93 (95% CI: 491.18–584.68) in 2030. The widening 95% confidence intervals reflect increasing uncertainty over longer-term forecasts. Qatar shows a mild decrease, from 347.60 (95% CI: 346.24–348.97) in 2022 to 341.84 (95% CI: 319.74–363.94) in 2030, suggesting a relatively stable burden with slight reductions.For Saudi Arabia, DALYs are projected to decline steadily from 519.69 (95% CI: 518.47–520.91) in 2022 to 439.73 (95% CI: 395.15–484.31) in 2030, indicating a notable reduction in disease burden. The United Arab Emirates also shows a strong decreasing trend, from 333.02 (95% CI: 332.16–333.89) in 2022 to 298.43 (95% CI: 267.69–329.17) in 2030, although the wider intervals toward the end of the period reflect moderate long-term uncertainty. Table 2

**Table 2:** ARIMA Forecast of Blindness Prevalence in GCC Countries, 2022–2030.

Country	Year	Point_Forecast	Lo_80	Hi_80	Lo_95	Hi_95
Bahrain	2022	348.0586	347.7373	348.3799	347.5672	348.55
	2023	346.2672	345.4452	347.0891	345.0101	347.5243
	2024	344.3913	342.9184	345.8642	342.1387	346.644
	2025	342.4428	340.2046	344.6809	339.0198	345.8657
	2026	340.4283	337.3369	343.5197	335.7004	345.1562
	2027	338.3526	334.3388	342.3664	332.2141	344.4911
	2028	336.2194	331.2284	341.2103	328.5863	343.8524
	2029	334.0318	328.0197	340.0439	324.8371	343.2265
	2030	331.7929	324.7243	338.8616	320.9824	342.6035
Kuwait	2022	333.6686	333.2927	334.0446	333.0936	334.2436
	2023	331.8155	330.77	332.861	330.2166	333.4144
	2024	329.8805	327.994	331.767	326.9954	332.7656
	2025	327.8933	325.1808	330.6057	323.745	332.0416
	2026	325.8729	322.3866	329.3591	320.5411	331.2046
	2027	323.8313	319.6305	328.032	317.4068	330.2558
	2028	321.7763	316.9176	326.6349	314.3456	329.2069
	2029	319.7126	314.2469	325.1784	311.3535	328.0718
	2030	317.6436	311.6151	323.6721	308.4238	326.8633
Oman	2022	523.7378	522.948	524.5277	522.5299	524.9458
	2023	524.1052	521.7746	526.4358	520.5409	527.6695
	2024	525.1925	520.5291	529.856	518.0604	532.3247
	2025	526.8239	519.0866	534.5611	514.9908	538.657
	2026	528.8335	517.3708	540.2963	511.3027	546.3643
	2027	531.071	515.342	546.8001	507.0155	555.1265
	2028	533.4043	512.9889	553.8198	502.1816	564.6271
	2029	535.7219	510.3217	561.122	496.8757	574.5681
	2030	537.9331	507.366	568.5001	491.1848	584.6813
Qatar	2022	347.6039	346.7129	348.4949	346.2412	348.9666
	2023	346.8494	344.8676	348.8313	343.8184	349.8804
	2024	346.1049	342.8054	349.4044	341.0587	351.151
	2025	345.3702	340.5643	350.176	338.0202	352.7201
	2026	344.6451	338.1702	351.12	334.7426	354.5477
	2027	343.9296	335.642	352.2172	331.2548	356.6045
	2028	343.2236	332.9944	353.4527	327.5794	358.8677
	2029	342.5268	330.2393	354.8143	323.7347	361.3189
	2030	341.8392	327.3865	356.292	319.7357	363.9428
Saudi Arabia	2022	519.687	518.8904	520.4835	518.4687	520.9052
	2023	510.8144	508.0913	513.5375	506.6498	514.979
	2024	501.3084	495.5349	507.0819	492.4786	510.1381
	2025	491.5141	482.0479	500.9804	477.0367	505.9915
	2026	481.4831	468.0846	494.8815	460.9919	501.9742
	2027	471.2575	453.853	488.6621	444.6396	497.8755
	2028	460.8723	439.4764	482.2681	428.1501	493.5944
	2029	450.3558	425.0345	475.677	411.6303	489.0813
	2030	439.7315	410.5807	468.8824	395.1492	484.3139
United Arab Emirates	2022	333.0219	332.4575	333.5863	332.1588	333.8851
	2023	329.9968	328.3544	331.6392	327.4849	332.5087
	2024	326.4579	323.2122	329.7036	321.4941	331.4217
	2025	322.4761	317.153	327.7992	314.3351	330.6171
	2026	318.1253	310.3232	325.9274	306.193	330.0576
	2027	313.4786	302.8779	324.0792	297.2663	329.6909
	2028	308.6053	294.9702	322.2405	287.7522	329.4585
	2029	303.5691	286.7433	320.3949	277.8363	329.3019
	2030	298.4261	278.3259	318.5264	267.6854	329.1669

The ARIMA models fitted to historical blindness DALY data in Gulf countries demonstrate adequate fit and satisfactory model performance. In Bahrain, an ARIMA(2,1,0) model was selected, with relatively low AIC (6.56) and BIC (12.29) values, and the residuals were stationary with a Ljung-Box p-value of 0.60, indicating no significant autocorrelation. The RMSE (0.23) and MAE (0.20) suggest good predictive accuracy. Kuwait’s data were modeled with an ARIMA(1,1,2) specification, showing low error metrics (RMSE = 0.27, MAE = 0.22) and a Ljung-Box p-value of 0.78, indicating well-behaved residuals. United Arab Emirates used an ARIMA(2,1,0) model, with slightly higher RMSE (0.41) and MAE (0.35), but residuals remained stationary and uncorrelated (Ljung-Box p = 0.40). For Oman, the ARIMA(2,1,0) model had higher RMSE (0.59) and MAE (0.47), reflecting greater variability in the data, though residuals were stationary. Saudi Arabia’s ARIMA (1,1,3) model showed moderate fit (RMSE = 0.56, MAE = 0.42) with stationary residuals (p = 0.69), and Qatar’s ARIMA(1,1,0) model had the highest RMSE (0.67) and MAE (0.49), yet residuals were also stationary. Table 3

**Table 3:** Parameters of ARIMA forecasting.

Country	ARIMA Order	BIC	RMSE	MAE	Ljung_Box_p	Stationary Residuals	Forecast Horizon
Bahrain	(2,1,0)	6.56	12.29	0.23	0.20	Yes	2022-2030
Kuwait	(1,1,2)	18.37	25.54	0.27	0.22	Yes	2022-2030
United Arab Emirates	(2,1,0)	45.71	51.45	0.41	0.35	Yes	2022-2030
Oman	(2,1,0)	66.27	70.57	0.59	0.47	Yes	2022-2030
Saudi Arabia	(1,1,3)	69.00	77.60	0.56	0.42	Yes	2022-2030
Qatar	(1,1,0)	71.60	74.47	0.67	0.49	Yes	2022-2030

**Fig 4:**
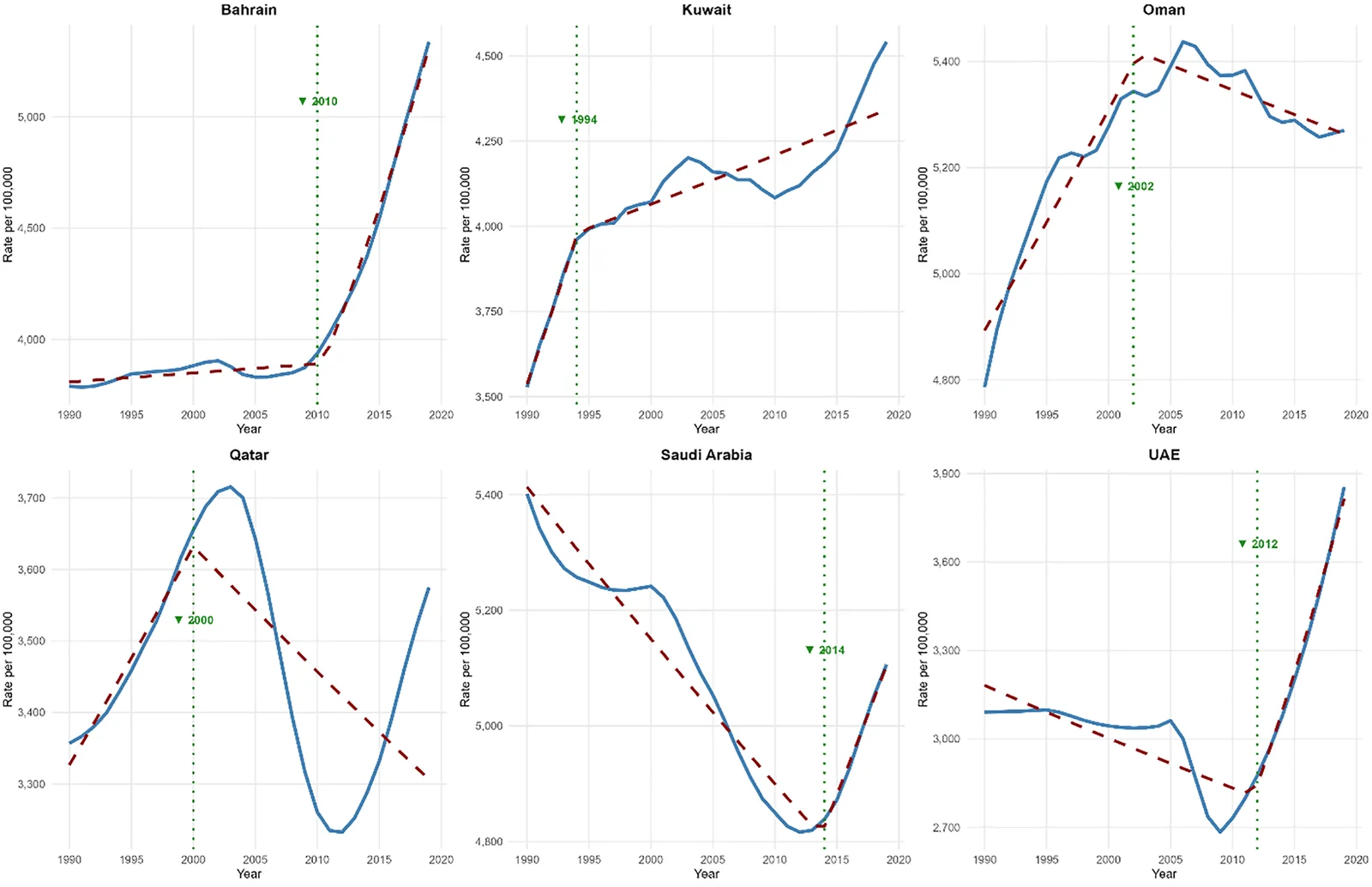
Temporal trends and inflection points in blindness prevalence within the Gulf Cooperation Council (GCC): A Joinpoint analysis.

The joinpoint regression analysis identified statistically significant breakpoints in age-standardized blindness prevalence across all six GCC countries between 1990 and 2021 (permutation test p < 0.05 for each country). The temporal dynamics revealed distinct regional patterns across the region (Fig 4).

Oman exhibited a significant joinpoint in 2002 (APC: + 0.82%, 95% CI: 0.63 to 1.01, p < 0.001), marking an initial increasing trend from 1990 to 2002, followed by a successful transition to a significant annual decline in the post-2002 period (APC: −0.15%, 95% CI: −0.22 to −0.08, p < 0.001). This represented the region’s most substantial reduction in blindness prevalence.

Qatar demonstrated a joinpoint in 2000, with an initial increase from 1990 to 2000 (APC: + 0.88%, 95% CI: 0.77 to 1.00, p < 0.001), followed by a significant decline thereafter (APC: −0.50%, 95% CI: −0.85 to −0.16, p = 0.007).

Bahrain showed a joinpoint in 2010, with a modest increase from 1990 to 2010 (APC: + 0.11%, 95% CI: 0.05 to 0.17, p = 0.001), followed by a marked acceleration in prevalence increase post-2010 (APC: + 3.54%, 95% CI: 3.23 to 3.85, p < 0.001), indicating a concerning reversal of the earlier stable trend.

Kuwait demonstrated a joinpoint in 1994, with a sharp increase from 1990 to 1994 (APC: + 2.94%, 95% CI: 2.63 to 3.25, p < 0.001), followed by a sustained but slower increase thereafter (APC: + 0.35%, 95% CI: 0.25 to 0.46, p < 0.001), suggesting ongoing but decelerating burden.

Saudi Arabia exhibited a unique pattern with a joinpoint in 2014. The kingdom experienced a significant decline from 1990 to 2014 (APC: −0.49%, 95% CI: −0.55 to −0.43, p < 0.001), followed by a statistically significant reversal and sharp increase in the post-2014 period (APC: + 1.12%, 95% CI: 1.00 to 1.25, p < 0.001), representing the most concerning trend reversal in the region.

The United Arab Emirates showed a joinpoint in 2012, with a robust downward trend from 1990 to 2012 (APC: −0.55%, 95% CI: −0.73 to −0.37, p < 0.001), followed by a dramatic reversal with a sharp increase post-2012 (APC: + 4.28%, 95% CI: 3.96 to 4.61, p < 0.001), indicating the most rapid recent increase in blindness prevalence among all GCC countries. Table 4

**Table 4:** Annual Percent Change (APC) in Blindness Prevalence Across Gulf Cooperation Council Countries, 1990–2019: Joinpoint Regression Analysis.

Country	Period	APC (%)	95% CI	p-value
Bahrain	1990–2010	0.11	0.05 to 0.17	0.002
	2010–2019	3.54	3.23 to 3.85	<0.001
Kuwait	1990–1994	2.94	2.63 to 3.25	<0.001
	1994–2019	0.35	0.25 to 0.46	<0.001
Oman	1990–2002	0.82	0.63 to 1.01	<0.001
	2002–2019	−0.15	−0.22 to −0.08	<0.001
Qatar	1990–2000	0.88	0.77 to 1.00	<0.001
	2000–2019	−0.50	−0.85 to −0.16	0.007
Saudi Arabia	1990–2014	−0.49	−0.55 to −0.43	<0.001
	2014–2019	1.12	1.00 to 1.25	<0.001
UAE	1990–2012	−0.55	−0.73 to −0.37	<0.001
	2012–2019	4.28	3.96 to 4.61	<0.001

Note: APC = annual percent change; CI = confidence interval. Positive APC values indicate increasing blindness prevalence, whereas negative values indicate decreasing prevalence. All reported APCs are statistically significant at p < 0.05.

## Discussion

This study aimed to fill critical gaps in our understanding of blindness and vision loss trends within the GCC, focusing on the period from 1990 to 2021 and providing predictions for 2022–2030 through robust time-series analysis. Time series analysis was proven to be effective in prediction of different health outcomes.[18–20]

### Study main findings

The comprehensive analysis of blindness prevalence trends in GCC countries from 1990 to 2021, with forecasts up to 2030, reveals distinctive patterns across the studied countries. Oman and Qatar exhibit an upward trajectory in prevalence rates, emphasizing the growing burden of visual impairment over the observed years. Notably, gender differences are evident, with prevalence consistently higher among females. The DALYs due to blindness show a concerning decrease, with Saudi Arabia reporting the highest DALYs in 2021. The forecasted prevalence and DALYs for 2022–2030 suggests ongoing challenges.

### Interpretation of the main findings

In this study there is increasing prevalence blindness and vision loss in the GCC countries. In the same line, a study conducted in the eastern Mediterranean region found that Oman and Saudi Arabi reported the highest prevalence of vision loss. [21] It is worth noting that few studies assessed visual impairment in GCC countries. However, a different prevalence of blindness and vision loss was reported in these few studies. Al-Shaaln et al., [22] conducted a study on 620 Saudi adults aged 18 and older from Aljouf primary health care centers found that 13.9% of them had visual impairment. A lower prevalence was reported in UAE. A cross-sectional eye health study conducted in Dubai between 2019–2020 aimed to understand the prevalence, causes, and risk factors of visual impairment (VI) among 892 Emiratis and non-Emiratis citizens. The prevalence of mild, moderate, and severe VI was 4.7% for Emiratis and 3.6% for non-Emiratis. [23] A lower prevalence of VI at 4.0% and blindness at 0.8% was derived from a hospital-based study conducted in the city of Al-Ain, situated in the emirate of Abu Dhabi.[24]

Alabdulwahhab et al., [25] investigated the causes of vision impairment and blindness in schools in Qassim province, Saudi Arabia. A cross-sectional study found that retinitis pigmentosa (26%), optic atrophy (16%), glaucoma (7%), head trauma (6%), nystagmus (6%), retinopathy of prematurity (6%), ocular albinism (4%), corneal opacities (4%), amblyopia (3%) and other causes (22%) were the leading causes of disability. Another study conducted in Aljouf reported that the main medical causes were refractive errors (36.0%), followed by cataract (29.1%) and diabetic retinopathy (20.9%). [22]

### Gender-based differences

The current study found that there was female predominance through the study period. UAE had a reversed pattern with higher male prevalence. In the same line, Pandova et al, reported higher affection among females than males, [26] and in UAE [23].

**Impact of blindness and vision loss on quality of life**: In this blindness and vision loss had a great impact on the GCC countries. In a systematic review comprising 138 studies, which investigated vision impairment (VI) stemming from unspecified causes or seven prevalent global causes (cataract, uncorrected refractive error, diabetic retinopathy, glaucoma, age-related macular degeneration, corneal opacity, and trachoma), the average quality assessment score was determined to be 78%. [27] A study conducted in United Kingdom (UK) found that the direct costs within the health care system reached £3.0 billion, with inpatient and day care expenses accounting for £735 million (24.6%), and outpatient costs making up £771 million (25.8%). Additionally, indirect costs associated with these conditions amounted to £5.65 billion (ranging from £5.12 to £6.22 billion).[3]

### Implication of the study findings

This study has important implications for public health and policy in the Gulf Cooperation Council countries. The consistent rise in blindness prevalence highlights the urgent need for targeted interventions and public health planning. Given the observed disparities in prevalence between males and females, gender-specific strategies are required. The socio-cultural context plays a crucial role, emphasizing the importance of culturally sensitive interventions, awareness campaigns, and healthcare accessibility. Strengthening healthcare infrastructure is imperative to accommodate the increasing burden of visual impairment, necessitating improvements in facilities, workforce skills, and service accessibility. Policymakers should incorporate these findings into policy formulation, allocating resources for eye care programs and integrating eye health into primary healthcare systems. Continued monitoring and research are vital for refining forecasting models and assessing intervention effectiveness. International collaboration and community engagement are essential components of a comprehensive approach to tackle the complex challenges posed by the rising prevalence of blindness in the GCC region.

Joinpoint analysis identified notable temporal changes in blindness prevalence across all GCC countries. In Oman (2004) and Qatar (2000), trends shifted from rising to declining following the roll-out of WHO Vision 2020 and the expansion of primary eye care services, with a 3–5 year delay likely reflecting disease progression and the time needed for programs to take effect. Bahrain (2011), Kuwait (1994), and the UAE (2012) exhibited persistent downward trends, linked to cumulative investments in eye health infrastructure, with Kuwait’s earlier joinpoint suggesting earlier program initiation. In contrast, Saudi Arabia experienced a worrisome upturn after 2014 (APC: + 0.05%, p < 0.05), possibly associated with increasing diabetes burden [28,29], insufficient screening coverage in rapidly growing urban settings, or enhanced case detection [30].

### Strengths and limitations

This study has various strengths that add to its importance in evaluating visual impairment trends in the GCC countries. An extensive time period from 1990 to 2021 allows for a complete examination of long-term trends. In addition, forecasting capabilities for the years 2022–2030 increase the study’s practical value for future public health planning. The reliance on data from the GBD 2021 Study lends credibility to the findings. The methodology of the study is robust, utilizing time-series analysis techniques such as ARIMA models, and the validation process ensures that predictions are aligned with observed data. However, study is not without its limitations. Dependence on secondary data sources introduces potential biases, as the accuracy of findings hinges on the quality of data from the GBD 2021 Study and other publications. Because of differences in healthcare systems, socioeconomic conditions, and cultural factors, generalization of results beyond the GCC countries may be limited. The study’s modeling approach has inherent limitations, particularly in capturing sudden, unpredictable events, and assumptions about temporal changes in visual impairment prevalence may be influenced by the stability of risk factors over time. Despite efforts to validate predictions, forecasting uncertainties persist, and external factors not taken into account in the model may have an impact on future prevalence rates. Furthermore, the study does not evaluate the efficacy of specific interventions or policies, which limits insights into potential areas for improvement.

## Conclusions

In conclusion, this research provides a comprehensive examination of blindness prevalence trends in GCC countries from 1990 to 2021, with projections up to 2030. The consistent and substantial upward trends in blindness prevalence across the GCC highlight the urgent need for targeted public health interventions. Gender disparities, with varying prevalence patterns between males and females, emphasize the necessity for gender-specific healthcare strategies. Projections for 2020–2024 suggest ongoing challenges, with some countries anticipating a continued rise in prevalence. These findings collectively underscore the critical importance of proactive and culturally sensitive public health initiatives, improved healthcare infrastructure, and evidence-based policymaking to address the escalating burden of visual impairment in the GCC region. Continuous monitoring and international collaboration are essential for refining strategies and ensuring the effectiveness of interventions in the dynamic landscape of eye health.

## Supporting information

S1 File Parameter Sensitivity (Alternative ARIMA Specifications). (DOCX)
