# Supplementary material for: Understanding visual impairment trends in the Gulf Council Countries: An analysis from 1990 to 2021 and time-series predictions for 2030
Source: PLoS One. 2026 Sep 8;21(9):e0357032. doi: 10.1371/journal.pone.0357032 (PMC13552931; doi:10.1371/journal.pone.0357032)
Supplement: Supplementary file 1 [file pone.0357032.s001.docx]

**Parameter Sensitivity (Alternative ARIMA Specifications)**

We tested 11 alternative ARIMA(p,d,q) specifications around the optimal model identified by auto.arima(). The coefficient of variation (CV) for 2030 forecasts across alternative models was:

| Country | CV (%) | Interpretation |
| --- | --- | --- |
| Oman | 0.8% | Excellent stability |
| Kuwait | 4.1% | Good stability |
| Bahrain | 4.6% | Good stability |
| Qatar | 6.9% | Good stability |
| Saudi Arabia | 6.9% | Good stability |
| UAE | 13.4% | Moderate stability |

Forecasts for Oman, Kuwait, Bahrain, Qatar, and Saudi Arabia showed narrow ranges (<7% variation), confirming model robustness. The UAE showed wider variation (13.4%), reflecting greater structural uncertainty in its trend.

**Training Window Sensitivity**

We refit models using different historical training periods (20-year: 1990-2009; 25-year: 1990-2014; 30-year: 1990-2019) to assess temporal stability.

| Country | CV Across Windows (%) | Stability Assessment |
| --- | --- | --- |
| Oman | 0.3% | Excellent |
| Qatar | 4.3% | Excellent |
| Saudi Arabia | 8.4% | Good |
| UAE | 8.0% | Good |
| Kuwait | 13.7% | Moderate |
| Bahrain | 27.5% | Sensitive |

Oman, Qatar, and Saudi Arabia demonstrated remarkable stability across training windows. Bahrain exhibited high sensitivity (CV=27.5%), suggesting its trend is influenced by recent changes. Bahrain's forecasts should therefore be interpreted with greater caution and updated as new data become available.

**Benchmarking Against Alternative Models**

We compared our primary ARIMA models against Simple Exponential Smoothing (SES) and ETS (Error, Trend, Seasonality) frameworks using time-series cross-validation (k=5).

| Country | ARIMA MAPE (%) | SES MAPE (%) | ETS MAPE (%) | Best Model |
| --- | --- | --- | --- | --- |
| Saudi Arabia | 1.57 | 2.89 | 2.34 | ARIMA |
| Oman | 1.80 | 2.45 | 2.11 | ARIMA |
| Bahrain | 2.88 | 3.76 | 3.42 | ARIMA |
| Kuwait | 3.37 | 4.12 | 3.89 | ARIMA |
| Qatar | 3.50 | 4.33 | 3.95 | ARIMA |
| UAE | 4.49 | 5.28 | 4.86 | ARIMA |

ARIMA models consistently outperformed SES and ETS alternatives across all countries, with mean MAPE improvement ranging from 0.8% (UAE) to 2.3% (Saudi Arabia). All countries achieved MAPE below 5%, indicating acceptable forecast accuracy

**Overall Robustness Assessment**

| Country | Parameter Sensitivity | Training Window | Cross-Validation | Overall Reliability |
| --- | --- | --- | --- | --- |
| Oman | Excellent | Excellent | Excellent | Excellent |
| Saudi Arabia | Good | Good | Excellent | Excellent |
| Qatar | Good | Excellent | Good | Good |
| Kuwait | Good | Moderate | Good | Good |
| Bahrain | Good | Sensitive | Good | Moderate |
| UAE | Moderate | Good | Good | Moderate |

Forecasts for Oman and Saudi Arabia are highly robust. Qatar and Kuwait show good reliability. Bahrain and UAE projections should be interpreted with greater caution, with Bahrain requiring updated validation as new data emerge. The consistent performance across all sensitivity analyses substantially strengthens confidence in our primary forecasts for the GCC region from 2022 to 2030.
