# Supplementary material for: Understanding visual impairment trends in the Gulf Council Countries: An analysis from 1990 to 2021 and time-series predictions for 2030
Source: PLoS One. 2026 Sep 8;21(9):e0357032. doi: 10.1371/journal.pone.0357032 (PMC13552931; doi:10.1371/journal.pone.0357032)
Supplement: Supplementary file 3 [file pone.0357032.s003.docx]

**Responses to reviewers**

PONE-D-25-28331

Editorial comments

“Understanding Visual Impairment Trends in the Gulf Council Countries: An Analysis from 1990 to 2019 and Time-Series Predictions for 2020-2024”.

1. Please ensure that your manuscript meets PLOS ONE's style requirements, including those for file naming.

Response: Thank you for your message. I reviewed the manuscript and ensured that it fully complies with PLOS ONE's style requirements, including the guidelines for file naming.

2. PLOS requires an ORCID iD for the corresponding author in Editorial Manager on papers submitted after December 6th, 2016. Please ensure that you have an ORCID iD and that it is validated in Editorial Manager. To do this, go to ‘Update my Information’ (in the upper left-hand corner of the main menu), and click on the Fetch/Validate link next to the ORCID field. This will direct you to the ORCID site, where you can create a new ID or authenticate an existing ID in Editorial Manager.

Response: I ensured that my ORCID iD is properly validated in Editorial Manager by following the steps provided.

Please complete your Competing Interests on the online submission form to state any Competing Interests. If you have no competing interests, please state ""The authors have declared that no competing interests exist."", as detailed online in our guide for authors at <http://journals.plos.org/plosone/s/submit-now>

Response: I completed the Competing Interests section in the online submission form as instructed. As no competing interests exist, I included the statement: "The authors have declared that no competing interests exist.

This information should be included in your cover letter; we will change the online submission form on your behalf.

4. Please provide a complete Data Availability Statement in the submission form, ensuring you include all necessary access information or a reason for why you are unable to make your data freely accessible. If your research concerns only data provided within your submission, please write "All data are in the manuscript and/or supporting information files" as your Data Availability Statement.

Response: Thank you for the reminder. I provided a complete Data Availability Statement in the submission form, including all necessary access details. If applicable, I included the statement: All data are in the manuscript and/or supporting information files.

5. Please amend either the abstract on the online submission form (via Edit Submission) or the abstract in the manuscript so that they are identical.

Response: Thank you for the notice. I amended the abstract to ensure it is identical in both the submission form and the manuscript.

6. If the reviewer comments include a recommendation to cite specific previously published works, please review and evaluate these publications to determine whether they are relevant and should be cited. There is no requirement to cite these works unless the editor has indicated otherwise.

Response: Thank you for the clarification. I carefully reviewed the suggested references and assessed their relevance to the manuscript. Citations included where appropriate, in line with the editor’s guidance.

Reviewers' comments:

- Reviewer #1: Considering the methodology and results presented in this study, several methodological and analytical issues can be noted that may affect the strength of the conclusions and reliability of the predictive models. The first of these concerns relates to the use of data dating back only to 2019, while the GBD database provides updated data up to 2021 or later. Failure to utilize this more recent data weakens the accuracy of the forecasts made for the period from 2020 to 2024 and reduces the credibility of the future trends presented. It is highly recommended to update the data used to include the most recent available years, as this would enhance reader confidence in the forecast results.
- We appreciate the reviewer’s valuable observation regarding the use of the Global Burden of Disease (GBD) dataset. We would like to clarify that the Institute for Health Metrics and Evaluation (IHME) has not released GBD 2023 recent estimates. More recent data are not publicly available. Consequently, our analysis relied on the most up-to-date dataset **[up to 2021]** and a comprehensive dataset accessible at the time of conducting this study.
- The results have been revised to reflect the findings of recent studies published in 2021.
- Given that we are in 2025, it is essential that forecasts are not limited to 2024 alone, but rather provide independent future estimates extending several years into the future, such as 2030, so that the study is more useful to decision-makers and can be effectively used in long-term health planning across the Gulf countries.
- We thank the reviewer for this important suggestion regarding extending the forecast horizon. Our initial analysis was restricted to 2020–2024 in order to provide short-term and more immediately applicable estimates for decision-making. However, we agree that long-term projections (e.g., to 2030) would add significant value for strategic health planning in the Gulf countries. To address this, we have extended our forecasts to 2030 using the available dataset, while clearly noting the assumptions and limitations associated with longer-term projections. This addition will allow policymakers to better align with regional and global health targets, including the Sustainable Development Goals (SDGs).
- On the other hand, the study did not sufficiently explain how the most appropriate ARIMA model was identified. Although the use of AIC, BIC, and ACF/PACF analysis is mentioned, the actual results of these criteria, as well as the chosen values for p, d, and q, are not provided. Details regarding reliability tests, such as Augmented Dickey-Fuller, are also missing, which are essential before applying any timescale model. This methodological ambiguity makes it difficult to assess the appropriateness of the model used.
- There is also a weakness in the analytical interpretation of the results. Many figures are presented regarding the prevalence of visual impairment and DALYs, but without adequate explanation for the significant differences between countries. For example, the potential reason behind the very high figures in Qatar compared to other countries, or the reasons for the fluctuations in the UAE's figures, are not explained. The study also does not indicate whether the differences in figures are statistically significant, a necessary element in any comparative analysis. Introducing statistical significance tests would improve the strength of the conclusions and strengthen the credibility of the analysis.

We thank the reviewer for these insightful comments on the methodology and analytical interpretation.

- Model selection and diagnostics: We agree that transparency in the model identification process is essential. In our revised manuscript, we have provided detailed information on the criteria used to identify the most appropriate ARIMA model. Specifically, we now report the candidate models tested, their corresponding AIC and BIC values, and the justification for selecting the final model. We also added the chosen values for p, d, and q and included the results of the Augmented Dickey-Fuller (ADF) test to demonstrate stationarity prior to model application. Furthermore, diagnostic checks (residual analysis, ACF/PACF plots of residuals, and Ljung–Box test results) have been added to ensure the reliability of the selected model.
- Analytical interpretation and cross-country differences: We acknowledge the reviewer’s point that the interpretation of country-specific trends requires more in-depth discussion. In the revised version, we have expanded the results and discussion to provide possible explanations for the observed differences. For example, we discuss how demographic structures, health system capacities, screening policies, and reporting practices may contribute to the relatively high figures in Qatar and the fluctuations seen in the UAE.
- Statistical significance of differences: We agree that reporting statistical significance enhances the robustness of comparative analysis. Accordingly, we have included statistical testing (where appropriate) to assess whether differences between countries are significant. These results are now clearly indicated in the text and tables.
- Furthermore, the steps taken to clean the data or deal with missing or outlier values are not clear. The use of Excel and R in the analysis was mentioned, but the cleaning methodology or transformations performed on the variables were not explained. This lack of detail makes the study difficult to reproduce, which contradicts the principles of scientific transparency. A detailed description of how the data were processed would be very helpful.
- We thank the reviewer for raising this important issue related to data cleaning and reproducibility. In the revised manuscript, we have added a detailed description of the data preprocessing steps undertaken prior to analysis. Specifically, we now explain how missing values were handled, the criteria used to identify and address outliers, and any transformations applied to the variables (e.g., logarithmic transformations for normalization, differencing for stationarity). We also clarified the role of Excel (for preliminary organization of raw data) and R (for statistical analysis, model fitting, and diagnostic testing).
- Another underestimated aspect is the lack of a meta-analysis across the Gulf countries. Each country was considered separately, without providing a comprehensive regional overview or a common summary highlighting the burden of vision impairment at the regional level. Including a meta-analysis or a regional analysis would enhance the practical impact of the study, especially for decision-makers and regional health policymakers.
- We appreciate the reviewer’s valuable suggestion regarding the inclusion of a meta-analysis or regional summary. While we recognize the importance of a comprehensive Gulf-wide overview, our primary objective in this study was to generate country-specific forecasts to reflect the heterogeneity of burden and trends within individual Gulf countries. Conducting a formal meta-analysis was beyond the scope of the current work, particularly as the forecasts were based on secondary GBD data rather than primary studies with pooled effect sizes.
- The study also did not conduct any **sensitivity analysis**, which is essential when making future projections. The absence of this type of analysis makes it difficult to assess the stability of the presented projections, or to determine the extent to which the model is affected by any changes in inputs or assumptions. Including a sensitivity analysis would help assess the reliability of the results and ensure they are not simply a reflection of limited training data. It is noted that the presentation of the results lacks organization, as there is a great deal of repetition in the description and insufficient use of illustrative tables or graphs, which could summarize the information and present it in a more effective and easier-to-understand manner. The results section should be reorganized and presented in a consolidated and comparative manner.
- We agree that sensitivity analysis is an important step in validating projections. Although a full sensitivity analysis was not included in the original submission, we acknowledge the need to assess the robustness of our forecasts. In the revised manuscript, we have performed a sensitivity analysis by varying key ARIMA parameters within reasonable ranges and examining the resulting projections. This analysis confirmed that the overall trends remain consistent, even when assumptions are modified. The details and results of this analysis have been added as supplementary material to enhance transparency.
- We also appreciate the reviewer’s observation regarding the organization and clarity of the results section. To address this, we have substantially revised the presentation of our findings. Specifically, we have reduced repetitive text, consolidated results across countries into comparative tables, and added summary graphs to highlight trends and differences more effectively. This restructuring makes the results clearer, more concise, and easier for readers—especially policymakers—to interpret.

All supplementary files have been uploaded and can be accessed via the following link, where all relevant materials are available:

<https://drive.google.com/drive/folders/1M7yTSnqUg0m-Pv5rfprC3X1_RwkQIjkw>
