# Supplementary material for: Understanding visual impairment trends in the Gulf Council Countries: An analysis from 1990 to 2021 and time-series predictions for 2030
Source: PLoS One. 2026 Sep 8;21(9):e0357032. doi: 10.1371/journal.pone.0357032 (PMC13552931; doi:10.1371/journal.pone.0357032)
Supplement: Supplementary file 4 [file pone.0357032.s004.docx]

**Reviewer Comments**

1. As pointed out by the previous reviewer, estimates based on 2019 GBD data is a methodological limitation and negates the timeliness of the article.

Since updated estimates from the Global Burden of Disease (GBD) study are now available for download, the authors may consider reanalyzing the data using the most recent dataset. Incorporating the latest estimates would improve the timeliness and relevance of the findings, as publications are generally strengthened when analyses are based on the most up-to-date data available.

2. Confirm the forecasting period in the title : is it 2024 or 2030. The paper reports projections for the year 2030
3. Introduction is very limited in providing background data regarding visual impairment in the gulf region. Provide more details and an overview into the policy and programs existing in the region to combat the burden
4. Jointpoint regression analysis could be carried out to see periods of significant change (acceleration, decline or stagnation)in the trend estimates of VISUAL IMPAIRMENT and the possible reasons could be explained in the discussion section
5. What assumptions were made when carrying out the prediction modelling?
6. What are the findings of the sensitivity analysis? No description has been provided.
