# Supplementary material for: Understanding visual impairment trends in the Gulf Council Countries: An analysis from 1990 to 2021 and time-series predictions for 2030
Source: PLoS One. 2026 Sep 8;21(9):e0357032. doi: 10.1371/journal.pone.0357032 (PMC13552931; doi:10.1371/journal.pone.0357032)
Supplement: Supplementary file 5 [file pone.0357032.s005.docx]

**Reviewer Comments**

As pointed out by the previous reviewer, estimates based on 2019 GBD data are methodological limitations and negates the timeliness of the article. Since updated estimates from the Global Burden of Disease (GBD) study are now available for download, the authors may consider reanalyzing the data using the most recent dataset. Incorporating the latest estimates would improve the timeliness and relevance of the findings, as publications are generally strengthened when analyses are based on the most up-to-date data available.

Thank you for this important comment. We agree that using the most up-to-date estimates from the Global Burden of Disease (GBD) study can enhance the timeliness and relevance of findings. However, the present study is based on a multi-country analysis including five countries. According to the most recent data access regulations of the Global Burden of Disease (GBD) study, downloading and using updated datasets for multinational analyses is currently restricted and not feasible at this level of aggregation. Therefore, reanalysis using the latest dataset could not be undertaken within the scope of this study.

We would also like to note that we have communicated with the journal editor regarding this issue. The editor acknowledged the constraint and advised us to proceed with submission of the revised manuscript without incorporating updated GBD estimates. To address this limitation transparently, we have clarified this point in the manuscript and acknowledged the use of 2019 GBD data as a limitation, while emphasizing that the analytical approach and comparative insights across countries remain valid and informative. We appreciate the reviewer’s suggestion and have reflected it in the discussion section.

Confirm the forecasting period in the title : is it 2024 or 2030. The paper reports projections for the year 2030

Thank you for pointing this out. We confirm that the forecasting period extends to the year 2030. The mention of 2024 in the title was a typographical error. The title has been revised accordingly to accurately reflect the projection period up to 2030.

Introduction is very limited in providing background data regarding visual impairment in the gulf region. Provide more details and an overview of the policy and programs existing in the region to combat the burden. We thank the reviewer for this insightful suggestion. We agree that providing a more comprehensive overview of the regional policy landscape strengthens the background of the study. We have added a new paragraph to the Introduction that details: The implementation of integrated Primary Eye Care (PEC) models in countries like Oman and Saudi Arabia. The collaborative role of regional bodies such as the Prevention of Blindness Union (PBU) and MEACO. The alignment of eye health goals with national transformative strategies, specifically Saudi Vision 2030 and the UAE National Agenda. The focus on shifting from communicable disease control to managing NCD-related vision loss, such as diabetic retinopathy.

[To reduce the growing burden of vision impairment, GCC member states have adopted strong national health strategies and regional collaborative frameworks [11]. Since the 1990s, countries such as Oman and Saudi Arabia have pioneered integrated primary eye care (PEC) models, in which vision screening is routinely included in primary healthcare services [12]. At the regional level, the Prevention of Blindness Union (PBU) and the Middle East Africa Council of Ophthalmology (MEACO)—often under Saudi leadership—have played a key role in coordinating “Vision 2020: The Right to Sight” initiatives across the Gulf. In addition, the healthcare reform objectives of Saudi Vision 2030 and the UAE National Agenda have increasingly prioritized preventive care, with particular attention to the early detection of diabetic retinopathy and glaucoma, both of which are highly prevalent in the region due to rising metabolic risk factors [11,13,14]. These efforts are further reinforced by the Gulf Cooperation Council Forum on Avoidable Blindness, which provides a platform for harmonizing clinical guidelines and allocating resources to meet the specific epidemiological needs of Gulf populations [15].]

Jointpoint regression analysis could be carried out to see periods of significant change (acceleration, decline or stagnation)in the trend estimates of VISUAL IMPAIRMENT and the possible reasons could be explained in the discussion section.

We thank the reviewer for this excellent suggestion. We have conducted joinpoint regression analysis and incorporated the findings into both the Methodology and Results sections.

**Methodology Addition:**

**Joinpoint Regression Analysis:**

Methdology

To identify significant temporal breakpoints in blindness prevalence trends, we conducted joinpoint regression analysis using the segmented package in R version 4.3.2. This method fits log-linear segmented regression models to the time-series data, allowing detection of statistically significant joinpoints where trends change direction or magnitude. We tested for 0, 1, and up to 2 joinpoints over the study period (1990-2021), with the optimal number selected using permutation tests (α = 0.05) with Bonferroni correction. For each identified segment, the Annual Percentage Change (APC) was calculated as APC = (e^β - 1) × 100, where β represents the segment-specific slope coefficient. Minimum segment length was restricted to three years to ensure model stability.

Results

The joinpoint regression analysis identified statistically significant breakpoints in age-standardized blindness prevalence across all six GCC countries between 1990 and 2021 (permutation test p < 0.05 for each country). The temporal dynamics revealed distinct regional patterns, most notably in Oman and Qatar, where inflection points in 2004 and 2000, respectively, marked a successful transition from increasing prevalence to significant annual declines. Following these joinpoints, Oman achieved the region's most substantial reduction with an APC of -0.12% (p < 0.001), while Qatar followed with an APC of -0.10% (p < 0.05). Bahrain and Kuwait demonstrated sustained progress; Bahrain maintained a consistent downward trajectory across both segments, whereas Kuwait shifted from a stable trend to a significant decline after 1994 (APC: -0.06%, p < 0.01). Saudi Arabia exhibited a unique and statistically significant rebound; after a long-term decline from 1990 to 2014, prevalence rates began to rise significantly in the post-2014 segment (APC: +0.05%, p < 0.05). Similarly, the UAE’s robust downward trend (APC: -0.12%) halted after its 2012 joinpoint, transitioning to a stable phase with no significant further reduction (APC: +0.02%, p = 0.218). Figure 4

**Figure 4: Temporal trends and inflection points in blindness prevalence within the Gulf Cooperation Council (GCC): A Joinpoint analysis.**

Discussion

Joinpoint analysis identified notable temporal changes in blindness prevalence across all GCC countries. In Oman (2004) and Qatar (2000), trends shifted from rising to declining following the roll-out of WHO Vision 2020 and the expansion of primary eye care services, with a 3–5 year delay likely reflecting disease progression and the time needed for programs to take effect. Bahrain (2011), Kuwait (1994), and the UAE (2012) exhibited persistent downward trends, linked to cumulative investments in eye health infrastructure, with Kuwait’s earlier joinpoint suggesting earlier program initiation. In contrast, Saudi Arabia experienced a worrisome upturn after 2014 (APC: +0.05%, p<0.05), possibly associated with increasing diabetes burden [29] [30], insufficient screening coverage in rapidly growing urban settings, or enhanced case detection [31].

What assumptions were made when carrying out the prediction modelling?

Our ARIMA forecasting models operated under eight explicit assumptions:

- Stationarity: The time series becomes stationary after appropriate differencing. This was verified using Augmented Dickey-Fuller (ADF) and KPSS tests (p < 0.05 after differencing for all country-specific series).
- Linearity: Past values have a linear relationship with future values. This implies that nonlinear dynamics (e.g., threshold effects, regime changes) are not captured by the model.
- Error Independence: Model residuals are uncorrelated (no autocorrelation). This was validated using Ljung-Box tests (all p > 0.05).
- Normality of Residuals: Residuals follow a normal distribution. This was confirmed by Shapiro-Wilk tests and Q-Q plots.
- Parameter Constancy: Model parameters remain stable over the forecast horizon (2022-2030). This implies that structural breaks, policy changes, or epidemiological shifts after 2021 are not accounted for by the model.
- No External Shocks: Future events (pandemics, wars, economic crises) do not alter baseline trends. The COVID-19 pandemic (2020-2021) was included in training data, but long-term effects remain uncertain.
- Data Quality: GBD 2021 estimates accurately represent true population prevalence. We accept that systematic biases in GBD modeling propagate to our forecasts.
- Homoscedasticity: Residual variance remains constant over time. This was confirmed by visual inspection of residual plots showing no systematic patterns.

What are the findings of the sensitivity analysis? No description has been provided.

We thank the reviewer for identifying this omission. Below are the comprehensive findings of our sensitivity analysis, which have been added to the Results section.

We conducted three complementary sensitivity analyses to assess the robustness and stability of our ARIMA forecasting models.

**Parameter Sensitivity (Alternative ARIMA Specifications)**

We tested 11 alternative ARIMA(p,d,q) specifications around the optimal model identified by auto.arima(). The coefficient of variation (CV) for 2030 forecasts across alternative models was:

| Country | CV (%) | Interpretation |
| --- | --- | --- |
| Oman | 0.8% | Excellent stability |
| Kuwait | 4.1% | Good stability |
| Bahrain | 4.6% | Good stability |
| Qatar | 6.9% | Good stability |
| Saudi Arabia | 6.9% | Good stability |
| UAE | 13.4% | Moderate stability |

Forecasts for Oman, Kuwait, Bahrain, Qatar, and Saudi Arabia showed narrow ranges (<7% variation), confirming model robustness. The UAE showed wider variation (13.4%), reflecting greater structural uncertainty in its trend.

**Training Window Sensitivity**

We refit models using different historical training periods (20-year: 1990-2009; 25-year: 1990-2014; 30-year: 1990-2019) to assess temporal stability.

| Country | CV Across Windows (%) | Stability Assessment |
| --- | --- | --- |
| Oman | 0.3% | Excellent |
| Qatar | 4.3% | Excellent |
| Saudi Arabia | 8.4% | Good |
| UAE | 8.0% | Good |
| Kuwait | 13.7% | Moderate |
| Bahrain | 27.5% | Sensitive |

Oman, Qatar, and Saudi Arabia demonstrated remarkable stability across training windows. Bahrain exhibited high sensitivity (CV=27.5%), suggesting its trend is influenced by recent changes. Bahrain's forecasts should therefore be interpreted with greater caution and updated as new data become available.

**Benchmarking Against Alternative Models**

We compared our primary ARIMA models against Simple Exponential Smoothing (SES) and ETS (Error, Trend, Seasonality) frameworks using time-series cross-validation (k=5).

| Country | ARIMA MAPE (%) | SES MAPE (%) | ETS MAPE (%) | Best Model |
| --- | --- | --- | --- | --- |
| Saudi Arabia | 1.57 | 2.89 | 2.34 | ARIMA |
| Oman | 1.80 | 2.45 | 2.11 | ARIMA |
| Bahrain | 2.88 | 3.76 | 3.42 | ARIMA |
| Kuwait | 3.37 | 4.12 | 3.89 | ARIMA |
| Qatar | 3.50 | 4.33 | 3.95 | ARIMA |
| UAE | 4.49 | 5.28 | 4.86 | ARIMA |

ARIMA models consistently outperformed SES and ETS alternatives across all countries, with mean MAPE improvement ranging from 0.8% (UAE) to 2.3% (Saudi Arabia). All countries achieved MAPE below 5%, indicating acceptable forecast accuracy

**Overall Robustness Assessment**

| Country | Parameter Sensitivity | Training Window | Cross-Validation | Overall Reliability |
| --- | --- | --- | --- | --- |
| Oman | Excellent | Excellent | Excellent | Excellent |
| Saudi Arabia | Good | Good | Excellent | Excellent |
| Qatar | Good | Excellent | Good | Good |
| Kuwait | Good | Moderate | Good | Good |
| Bahrain | Good | Sensitive | Good | Moderate |
| UAE | Moderate | Good | Good | Moderate |

Forecasts for Oman and Saudi Arabia are highly robust. Qatar and Kuwait show good reliability. Bahrain and UAE projections should be interpreted with greater caution, with Bahrain requiring updated validation as new data emerge. The consistent performance across all sensitivity analyses substantially strengthens confidence in our primary forecasts for the GCC region from 2022 to 2030.
