# Supplementary material for: Understanding visual impairment trends in the Gulf Council Countries: An analysis from 1990 to 2021 and time-series predictions for 2030
Source: PLoS One. 2026 Sep 8;21(9):e0357032. doi: 10.1371/journal.pone.0357032 (PMC13552931; doi:10.1371/journal.pone.0357032)
Supplement: Supplementary file 6 [file pone.0357032.s006.docx]

**1.** The results would be more informative if percentage changes, Annual Percentage Change (APC) or Average Annual Percentage Change (AAPC), together with their statistical significance, were presented alongside the absolute estimates.

**2.** The presentation of the joinpoint regression results requires further detail. Although APC values are reported for different trend segments, the corresponding 95% confidence intervals and exact *p*-values are not provided, making it difficult to assess the precision and statistical significance of the reported trends.

**3.** While the Methods section states that forecasts were generated with 95% prediction intervals, the Results primarily present point estimates without adequately conveying forecast uncertainty. Forecast figures should include 95% prediction intervals to illustrate the increasing uncertainty associated with longer-term projections. Presenting uncertainty bands graphically, along with summary tables of prediction intervals, would provide readers with a more realistic interpretation of the reliability and precision of the projected estimates.
