# Supplementary material for: Understanding visual impairment trends in the Gulf Council Countries: An analysis from 1990 to 2021 and time-series predictions for 2030
Source: PLoS One. 2026 Sep 8;21(9):e0357032. doi: 10.1371/journal.pone.0357032 (PMC13552931; doi:10.1371/journal.pone.0357032)
Supplement: Supplementary file 7 [file pone.0357032.s007.docx]

**Reply to Reviewer Comments**

**Manuscript Title:** Understanding Visual Impairment Trends in the Gulf Council Countries: An Analysis from 1990 to 2021 and Time-Series Predictions for 2030

**Reviewer Comment 1:**

*"The results would be more informative if percentage changes, Annual Percentage Change (APC) or Average Annual Percentage Change (AAPC), together with their statistical significance, were presented alongside the absolute estimates."*

**Response:**

We thank the reviewer for this valuable suggestion. We agree that presenting percentage changes and Annual Percentage Changes (APC) with statistical significance greatly enhances the interpretability of the results. In response, we have:

1. Calculated percentage changes for each country between 1990 and 2021 (relative change in prevalence rates).
2. Performed joinpoint regression analysis to calculate Annual Percentage Changes (APC) with 95% confidence intervals and exact p-values for each trend segment.
3. Created a comprehensive Table 3 that now includes:
   - 1990 and 2021 prevalence rates
   - Absolute and relative percentage changes
   - APC with 95% confidence intervals
   - p-values and significance indicators
   - Trend direction (increasing/decreasing/stable)

We believe these additions substantially enhance the clinical and epidemiological interpretability of our findings, allowing readers to better understand the magnitude and significance of temporal trends in blindness prevalence across the GCC region.

**Reviewer Comment 2:**

"The presentation of the joinpoint regression results requires further detail. Although APC values are reported for different trend segments, the corresponding 95% confidence intervals and exact p-values are not provided, making it difficult to assess the precision and statistical significance of the reported trends."

Response

We appreciate this important critique and have substantially enhanced our joinpoint regression analysis presentation. We have now:

Expanded the methodology section  to provide detailed description of the joinpoint regression approach, including:

Log-linear segmented regression model specification

Permutation test procedure for joinpoint selection (α = 0.05 with Bonferroni correction)

Minimum segment length restrictions (3 years)

APC calculation formula: APC = (e^β - 1) × 100

Created a new Table 3 that provides comprehensive joinpoint results including:

Country-specific joinpoint years

Segment periods

APC values for each segment with 95% confidence intervals

Exact p-values for each segment slope

Significance indicators with stars

Updated the Results section (pages 20-21) with detailed descriptions of each country's joinpoint findings, including:

Country: "Oman exhibited a significant joinpoint in 2002, with an initial increasing trend (APC: +0.82%, 95% CI: 0.63 to 1.01, p < 0.001), followed by a successful transition to significant annual decline (APC: -0.15%, 95% CI: -0.22 to -0.08, p < 0.001)."

Saudi Arabia: "The kingdom experienced a significant decline from 1990 to 2014 (APC: -0.49%, 95% CI: -0.55 to -0.43, p < 0.001), followed by a statistically significant reversal and sharp increase post-2014 (APC: +1.12%, 95% CI: 1.00 to 1.25, p < 0.001)."

UAE: "Robust downward trend from 1990 to 2012 (APC: -0.55%, 95% CI: -0.73 to -0.37, p < 0.001), followed by a dramatic reversal post-2012 (APC: +4.28%, 95% CI: 3.96 to 4.61, p < 0.001)."

These additions now allow readers to fully assess the precision, statistical significance, and clinical importance of the trend changes identified through joinpoint regression analysis.

**Reviewer Comment 3:**

"While the Methods section states that forecasts were generated with 95% prediction intervals, the Results primarily present point estimates without adequately conveying forecast uncertainty. Forecast figures should include 95% prediction intervals to illustrate the increasing uncertainty associated with longer-term projections. Presenting uncertainty bands graphically, along with summary tables of prediction intervals, would provide readers with a more realistic interpretation of the reliability and precision of the projected estimates."

**Response:**

We added table 2 showing the forecast and prediction interval as follows

**Table 2: ARIMA Forecast of Blindness Prevalence in GCC Countries, 2022–2030**

| Country | Year | Point_Forecast | Lo_80 | Hi_80 | Lo_95 | Hi_95 |
| --- | --- | --- | --- | --- | --- | --- |
| Bahrain | 2022 | 348.0586 | 347.7373 | 348.3799 | 347.5672 | 348.55 |
|  | 2023 | 346.2672 | 345.4452 | 347.0891 | 345.0101 | 347.5243 |
|  | 2024 | 344.3913 | 342.9184 | 345.8642 | 342.1387 | 346.644 |
|  | 2025 | 342.4428 | 340.2046 | 344.6809 | 339.0198 | 345.8657 |
|  | 2026 | 340.4283 | 337.3369 | 343.5197 | 335.7004 | 345.1562 |
|  | 2027 | 338.3526 | 334.3388 | 342.3664 | 332.2141 | 344.4911 |
|  | 2028 | 336.2194 | 331.2284 | 341.2103 | 328.5863 | 343.8524 |
|  | 2029 | 334.0318 | 328.0197 | 340.0439 | 324.8371 | 343.2265 |
|  | 2030 | 331.7929 | 324.7243 | 338.8616 | 320.9824 | 342.6035 |
| Kuwait | 2022 | 333.6686 | 333.2927 | 334.0446 | 333.0936 | 334.2436 |
|  | 2023 | 331.8155 | 330.77 | 332.861 | 330.2166 | 333.4144 |
|  | 2024 | 329.8805 | 327.994 | 331.767 | 326.9954 | 332.7656 |
|  | 2025 | 327.8933 | 325.1808 | 330.6057 | 323.745 | 332.0416 |
|  | 2026 | 325.8729 | 322.3866 | 329.3591 | 320.5411 | 331.2046 |
|  | 2027 | 323.8313 | 319.6305 | 328.032 | 317.4068 | 330.2558 |
|  | 2028 | 321.7763 | 316.9176 | 326.6349 | 314.3456 | 329.2069 |
|  | 2029 | 319.7126 | 314.2469 | 325.1784 | 311.3535 | 328.0718 |
|  | 2030 | 317.6436 | 311.6151 | 323.6721 | 308.4238 | 326.8633 |
| Oman | 2022 | 523.7378 | 522.948 | 524.5277 | 522.5299 | 524.9458 |
|  | 2023 | 524.1052 | 521.7746 | 526.4358 | 520.5409 | 527.6695 |
|  | 2024 | 525.1925 | 520.5291 | 529.856 | 518.0604 | 532.3247 |
|  | 2025 | 526.8239 | 519.0866 | 534.5611 | 514.9908 | 538.657 |
|  | 2026 | 528.8335 | 517.3708 | 540.2963 | 511.3027 | 546.3643 |
|  | 2027 | 531.071 | 515.342 | 546.8001 | 507.0155 | 555.1265 |
|  | 2028 | 533.4043 | 512.9889 | 553.8198 | 502.1816 | 564.6271 |
|  | 2029 | 535.7219 | 510.3217 | 561.122 | 496.8757 | 574.5681 |
|  | 2030 | 537.9331 | 507.366 | 568.5001 | 491.1848 | 584.6813 |
| Qatar | 2022 | 347.6039 | 346.7129 | 348.4949 | 346.2412 | 348.9666 |
|  | 2023 | 346.8494 | 344.8676 | 348.8313 | 343.8184 | 349.8804 |
|  | 2024 | 346.1049 | 342.8054 | 349.4044 | 341.0587 | 351.151 |
|  | 2025 | 345.3702 | 340.5643 | 350.176 | 338.0202 | 352.7201 |
|  | 2026 | 344.6451 | 338.1702 | 351.12 | 334.7426 | 354.5477 |
|  | 2027 | 343.9296 | 335.642 | 352.2172 | 331.2548 | 356.6045 |
|  | 2028 | 343.2236 | 332.9944 | 353.4527 | 327.5794 | 358.8677 |
|  | 2029 | 342.5268 | 330.2393 | 354.8143 | 323.7347 | 361.3189 |
|  | 2030 | 341.8392 | 327.3865 | 356.292 | 319.7357 | 363.9428 |
| Saudi Arabia | 2022 | 519.687 | 518.8904 | 520.4835 | 518.4687 | 520.9052 |
|  | 2023 | 510.8144 | 508.0913 | 513.5375 | 506.6498 | 514.979 |
|  | 2024 | 501.3084 | 495.5349 | 507.0819 | 492.4786 | 510.1381 |
|  | 2025 | 491.5141 | 482.0479 | 500.9804 | 477.0367 | 505.9915 |
|  | 2026 | 481.4831 | 468.0846 | 494.8815 | 460.9919 | 501.9742 |
|  | 2027 | 471.2575 | 453.853 | 488.6621 | 444.6396 | 497.8755 |
|  | 2028 | 460.8723 | 439.4764 | 482.2681 | 428.1501 | 493.5944 |
|  | 2029 | 450.3558 | 425.0345 | 475.677 | 411.6303 | 489.0813 |
|  | 2030 | 439.7315 | 410.5807 | 468.8824 | 395.1492 | 484.3139 |
| United Arab Emirates | 2022 | 333.0219 | 332.4575 | 333.5863 | 332.1588 | 333.8851 |
|  | 2023 | 329.9968 | 328.3544 | 331.6392 | 327.4849 | 332.5087 |
|  | 2024 | 326.4579 | 323.2122 | 329.7036 | 321.4941 | 331.4217 |
|  | 2025 | 322.4761 | 317.153 | 327.7992 | 314.3351 | 330.6171 |
|  | 2026 | 318.1253 | 310.3232 | 325.9274 | 306.193 | 330.0576 |
|  | 2027 | 313.4786 | 302.8779 | 324.0792 | 297.2663 | 329.6909 |
|  | 2028 | 308.6053 | 294.9702 | 322.2405 | 287.7522 | 329.4585 |
|  | 2029 | 303.5691 | 286.7433 | 320.3949 | 277.8363 | 329.3019 |
|  | 2030 | 298.4261 | 278.3259 | 318.5264 | 267.6854 | 329.1669 |
