# Supplementary material for: Understanding visual impairment trends in the Gulf Council Countries: An analysis from 1990 to 2021 and time-series predictions for 2030
Source: PLoS One. 2026 Sep 8;21(9):e0357032. doi: 10.1371/journal.pone.0357032 (PMC13552931; doi:10.1371/journal.pone.0357032)
Supplement: Peer review history

# Author comment: Author response to Decision Letter 0

**DOI:** 10.1371/journal.pone.0357032.r001

26 May 2025

Attachment Submitted filename: PLOSOne_Human_Subjects_Research_Checklist.docx

# Peer review: Decision Letter 0

**DOI:** 10.1371/journal.pone.0357032.r002

**Authors:** sunny narayan

11 Aug 2025

Dear Dr. Al zomia,

Thank you for submitting your manuscript to PLOS ONE. After careful consideration, we feel that it has merit but does not fully meet PLOS ONE’s publication criteria as it currently stands. Therefore, we invite you to submit a revised version of the manuscript that addresses the points raised during the review process.

Please submit your revised manuscript by Sep 25 2025 11:59PM. If you will need more time than this to complete your revisions, please reply to this message or contact the journal office at plosone@plos.org. When you're ready to submit your revision, log on to https://www.editorialmanager.com/pone/ and select the 'Submissions Needing Revision' folder to locate your manuscript file.

- A rebuttal letter that responds to each point raised by the academic editor and reviewer(s). You should upload this letter as a separate file labeled 'Response to Reviewers'.
- A marked-up copy of your manuscript that highlights changes made to the original version. You should upload this as a separate file labeled 'Revised Manuscript with Track Changes'.
- An unmarked version of your revised paper without tracked changes. You should upload this as a separate file labeled 'Manuscript'.

If you would like to make changes to your financial disclosure, please include your updated statement in your cover letter. Guidelines for resubmitting your figure files are available below the reviewer comments at the end of this letter.

If applicable, we recommend that you deposit your laboratory protocols in protocols.io to enhance the reproducibility of your results. Protocols.io assigns your protocol its own identifier (DOI) so that it can be cited independently in the future. For instructions see: https://journals.plos.org/plosone/s/submission-guidelines#loc-laboratory-protocols. Additionally, PLOS ONE offers an option for publishing peer-reviewed Lab Protocol articles, which describe protocols hosted on protocols.io. Read more information on sharing protocols at https://plos.org/protocols?utm_medium=editorial-email&utm_source=authorletters&utm_campaign=protocols.

We look forward to receiving your revised manuscript.

Kind regards,

sunny narayan

Academic Editor

PLOS ONE

Journal Requirements:

When submitting your revision, we need you to address these additional requirements.

1. Please ensure that your manuscript meets PLOS ONE's style requirements, including those for file naming. The PLOS ONE style templates can be found at

https://journals.plos.org/plosone/s/file?id=wjVg/PLOSOne_formatting_sample_main_body.pdf and

https://journals.plos.org/plosone/s/file?id=ba62/PLOSOne_formatting_sample_title_authors_affiliations.pdf.

2. PLOS requires an ORCID iD for the corresponding author in Editorial Manager on papers submitted after December 6th, 2016. Please ensure that you have an ORCID iD and that it is validated in Editorial Manager. To do this, go to ‘Update my Information’ (in the upper left-hand corner of the main menu), and click on the Fetch/Validate link next to the ORCID field. This will take you to the ORCID site and allow you to create a new iD or authenticate a pre-existing iD in Editorial Manager.

3. Thank you for stating the following in your Competing Interests section:

[NP].

Please complete your Competing Interests on the online submission form to state any Competing Interests. If you have no competing interests, please state ""The authors have declared that no competing interests exist."", as detailed online in our guide for authors at http://journals.plos.org/plosone/s/submit-now

This information should be included in your cover letter; we will change the online submission form on your behalf.

4. Please provide a complete Data Availability Statement in the submission form, ensuring you include all necessary access information or a reason for why you are unable to make your data freely accessible. If your research concerns only data provided within your submission, please write "All data are in the manuscript and/or supporting information files" as your Data Availability Statement.

5. Please amend either the abstract on the online submission form (via Edit Submission) or the abstract in the manuscript so that they are identical.

6. If the reviewer comments include a recommendation to cite specific previously published works, please review and evaluate these publications to determine whether they are relevant and should be cited. There is no requirement to cite these works unless the editor has indicated otherwise.

[Note: HTML markup is below. Please do not edit.]

Reviewers' comments:

Reviewer's Responses to Questions

**Comments to the Author**

1. Is the manuscript technically sound, and do the data support the conclusions?

Reviewer #1: Yes

**********

2. Has the statistical analysis been performed appropriately and rigorously?

Reviewer #1: No

**********

3. Have the authors made all data underlying the findings in their manuscript fully available?

The PLOS Data policy

Reviewer #1: Yes

**********

4. Is the manuscript presented in an intelligible fashion and written in standard English?

Reviewer #1: No

**********

Reviewer #1: Considering the methodology and results presented in this study, several methodological and analytical issues can be noted that may affect the strength of the conclusions and reliability of the predictive models. The first of these concerns relates to the use of data dating back only to 2019, while the GBD database provides updated data up to 2021 or later. Failure to utilize this more recent data weakens the accuracy of the forecasts made for the period from 2020 to 2024 and reduces the credibility of the future trends presented. It is highly recommended to update the data used to include the most recent available years, as this would enhance reader confidence in the forecast results.

Given that we are in 2025, it is essential that forecasts are not limited to 2024 alone, but rather provide independent future estimates extending several years into the future, such as 2030, so that the study is more useful to decision-makers and can be effectively used in long-term health planning across the Gulf countries.

On the other hand, the study did not sufficiently explain how the most appropriate ARIMA model was identified. Although the use of AIC, BIC, and ACF/PACF analysis is mentioned, the actual results of these criteria, as well as the chosen values for p, d, and q, are not provided. Details regarding reliability tests, such as Augmented Dickey-Fuller, are also missing, which are essential before applying any timescale model. This methodological ambiguity makes it difficult to assess the appropriateness of the model used.

There is also a weakness in the analytical interpretation of the results. Many figures are presented regarding the prevalence of visual impairment and DALYs, but without adequate explanation for the significant differences between countries. For example, the potential reason behind the very high figures in Qatar compared to other countries, or the reasons for the fluctuations in the UAE's figures, are not explained. The study also does not indicate whether the differences in figures are statistically significant, a necessary element in any comparative analysis. Introducing statistical significance tests would improve the strength of the conclusions and strengthen the credibility of the analysis.

Furthermore, the steps taken to clean the data or deal with missing or outlier values are not clear. The use of Excel and R in the analysis was mentioned, but the cleaning methodology or transformations performed on the variables were not explained. This lack of detail makes the study difficult to reproduce, which contradicts the principles of scientific transparency. A detailed description of how the data were processed would be very helpful.

Another underestimated aspect is the lack of a meta-analysis across the Gulf countries. Each country was considered separately, without providing a comprehensive regional overview or a common summary highlighting the burden of vision impairment at the regional level. Including a meta-analysis or a regional analysis would enhance the practical impact of the study, especially for decision-makers and regional health policymakers.

The study also did not conduct any sensitivity analysis, which is essential when making future projections. The absence of this type of analysis makes it difficult to assess the stability of the presented projections, or to determine the extent to which the model is affected by any changes in inputs or assumptions. Including a sensitivity analysis would help assess the reliability of the results and ensure they are not simply a reflection of limited training data. It is noted that the presentation of the results lacks organization, as there is a great deal of repetition in the description and insufficient use of illustrative tables or graphs, which could summarize the information and present it in a more effective and easier-to-understand manner. The results section should be reorganized and presented in a consolidated and comparative manner.

**********

what does this mean?). If published, this will include your full peer review and any attached files.

If you choose “no”, your identity will remain anonymous but your review may still be made public.

**Do you want your identity to be public for this peer review?**  For information about this choice, including consent withdrawal, please see our Privacy Policy

Reviewer #1: **Yes:** Suzan Abdel-Rahman

**********

[NOTE: If reviewer comments were submitted as an attachment file, they will be attached to this email and accessible via the submission site. Please log into your account, locate the manuscript record, and check for the action link "View Attachments". If this link does not appear, there are no attachment files.]

While revising your submission, please upload your figure files to the Preflight Analysis and Conversion Engine (PACE) digital diagnostic tool, https://pacev2.apexcovantage.com/. PACE helps ensure that figures meet PLOS requirements. To use PACE, you must first register as a user. Registration is free. Then, login and navigate to the UPLOAD tab, where you will find detailed instructions on how to use the tool. If you encounter any issues or have any questions when using PACE, please email PLOS at figures@plos.org. Please note that Supporting Information files do not need this step.

# Author comment: Author response to Decision Letter 1

**DOI:** 10.1371/journal.pone.0357032.r003

27 Sep 2025

Responses to reviewers

PONE-D-25-28331

Editorial comments

“Understanding Visual Impairment Trends in the Gulf Council Countries: An Analysis from 1990 to 2019 and Time-Series Predictions for 2020-2024”.

1. Please ensure that your manuscript meets PLOS ONE's style requirements, including those for file naming.

Response: Thank you for your message. I reviewed the manuscript and ensured that it fully complies with PLOS ONE's style requirements, including the guidelines for file naming.

2. PLOS requires an ORCID iD for the corresponding author in Editorial Manager on papers submitted after December 6th, 2016. Please ensure that you have an ORCID iD and that it is validated in Editorial Manager. To do this, go to ‘Update my Information’ (in the upper left-hand corner of the main menu), and click on the Fetch/Validate link next to the ORCID field. This will direct you to the ORCID site, where you can create a new ID or authenticate an existing ID in Editorial Manager.

Response: I ensured that my ORCID iD is properly validated in Editorial Manager by following the steps provided.

Please complete your Competing Interests on the online submission form to state any Competing Interests. If you have no competing interests, please state ""The authors have declared that no competing interests exist."", as detailed online in our guide for authors at http://journals.plos.org/plosone/s/submit-now

Response: I completed the Competing Interests section in the online submission form as instructed. As no competing interests exist, I included the statement: "The authors have declared that no competing interests exist.

This information should be included in your cover letter; we will change the online submission form on your behalf.

4. Please provide a complete Data Availability Statement in the submission form, ensuring you include all necessary access information or a reason for why you are unable to make your data freely accessible. If your research concerns only data provided within your submission, please write "All data are in the manuscript and/or supporting information files" as your Data Availability Statement.

Response: Thank you for the reminder. I provided a complete Data Availability Statement in the submission form, including all necessary access details. If applicable, I included the statement: All data are in the manuscript and/or supporting information files.

5. Please amend either the abstract on the online submission form (via Edit Submission) or the abstract in the manuscript so that they are identical.

Response: Thank you for the notice. I amended the abstract to ensure it is identical in both the submission form and the manuscript.

6. If the reviewer comments include a recommendation to cite specific previously published works, please review and evaluate these publications to determine whether they are relevant and should be cited. There is no requirement to cite these works unless the editor has indicated otherwise.

Response: Thank you for the clarification. I carefully reviewed the suggested references and assessed their relevance to the manuscript. Citations included where appropriate, in line with the editor’s guidance.

Reviewers' comments:

• Reviewer #1: Considering the methodology and results presented in this study, several methodological and analytical issues can be noted that may affect the strength of the conclusions and reliability of the predictive models. The first of these concerns relates to the use of data dating back only to 2019, while the GBD database provides updated data up to 2021 or later. Failure to utilize this more recent data weakens the accuracy of the forecasts made for the period from 2020 to 2024 and reduces the credibility of the future trends presented. It is highly recommended to update the data used to include the most recent available years, as this would enhance reader confidence in the forecast results.

• We appreciate the reviewer’s valuable observation regarding the use of the Global Burden of Disease (GBD) dataset. We would like to clarify that the Institute for Health Metrics and Evaluation (IHME) has not released GBD 2023 recent estimates. More recent data are not publicly available. Consequently, our analysis relied on the most up-to-date dataset [up to 2021] and a comprehensive dataset accessible at the time of conducting this study.

• The results have been revised to reflect the findings of recent studies published in 2021.

• Given that we are in 2025, it is essential that forecasts are not limited to 2024 alone, but rather provide independent future estimates extending several years into the future, such as 2030, so that the study is more useful to decision-makers and can be effectively used in long-term health planning across the Gulf countries.

• We thank the reviewer for this important suggestion regarding extending the forecast horizon. Our initial analysis was restricted to 2020–2024 in order to provide short-term and more immediately applicable estimates for decision-making. However, we agree that long-term projections (e.g., to 2030) would add significant value for strategic health planning in the Gulf countries. To address this, we have extended our forecasts to 2030 using the available dataset, while clearly noting the assumptions and limitations associated with longer-term projections. This addition will allow policymakers to better align with regional and global health targets, including the Sustainable Development Goals (SDGs).

• On the other hand, the study did not sufficiently explain how the most appropriate ARIMA model was identified. Although the use of AIC, BIC, and ACF/PACF analysis is mentioned, the actual results of these criteria, as well as the chosen values for p, d, and q, are not provided. Details regarding reliability tests, such as Augmented Dickey-Fuller, are also missing, which are essential before applying any timescale model. This methodological ambiguity makes it difficult to assess the appropriateness of the model used.

• There is also a weakness in the analytical interpretation of the results. Many figures are presented regarding the prevalence of visual impairment and DALYs, but without adequate explanation for the significant differences between countries. For example, the potential reason behind the very high figures in Qatar compared to other countries, or the reasons for the fluctuations in the UAE's figures, are not explained. The study also does not indicate whether the differences in figures are statistically significant, a necessary element in any comparative analysis. Introducing statistical significance tests would improve the strength of the conclusions and strengthen the credibility of the analysis.

We thank the reviewer for these insightful comments on the methodology and analytical interpretation.

• Model selection and diagnostics: We agree that transparency in the model identification process is essential. In our revised manuscript, we have provided detailed information on the criteria used to identify the most appropriate ARIMA model. Specifically, we now report the candidate models tested, their corresponding AIC and BIC values, and the justification for selecting the final model. We also added the chosen values for p, d, and q and included the results of the Augmented Dickey-Fuller (ADF) test to demonstrate stationarity prior to model application. Furthermore, diagnostic checks (residual analysis, ACF/PACF plots of residuals, and Ljung–Box test results) have been added to ensure the reliability of the selected model.

• Analytical interpretation and cross-country differences: We acknowledge the reviewer’s point that the interpretation of country-specific trends requires more in-depth discussion. In the revised version, we have expanded the results and discussion to provide possible explanations for the observed differences. For example, we discuss how demographic structures, health system capacities, screening policies, and reporting practices may contribute to the relatively high figures in Qatar and the fluctuations seen in the UAE.

• Statistical significance of differences: We agree that reporting statistical significance enhances the robustness of comparative analysis. Accordingly, we have included statistical testing (where appropriate) to assess whether differences between countries are significant. These results are now clearly indicated in the text and tables.

• Furthermore, the steps taken to clean the data or deal with missing or outlier values are not clear. The use of Excel and R in the analysis was mentioned, but the cleaning methodology or transformations performed on the variables were not explained. This lack of detail makes the study difficult to reproduce, which contradicts the principles of scientific transparency. A detailed description of how the data were processed would be very helpful.

• We thank the reviewer for raising this important issue related to data cleaning and reproducibility. In the revised manuscript, we have added a detailed description of the data preprocessing steps undertaken prior to analysis. Specifically, we now explain how missing values were handled, the criteria used to identify and address outliers, and any transformations applied to the variables (e.g., logarithmic transformations for normalization, differencing for stationarity). We also clarified the role of Excel (for preliminary organization of raw data) and R (for statistical analysis, model fitting, and diagnostic testing).

• Another underestimated aspect is the lack of a meta-analysis across the Gulf countries. Each country was considered separately, without providing a comprehensive regional overview or a common summary highlighting the burden of vision impairment at the regional level. Including a meta-analysis or a regional analysis would enhance the practical impact of the study, especially for decision-makers and regional health policymakers.

• We appreciate the reviewer’s valuable suggestion regarding the inclusion of a meta-analysis or regional summary. While we recognize the importance of a comprehensive Gulf-wide overview, our primary objective in this study was to generate country-specific forecasts to reflect the heterogeneity of burden and trends within individual Gulf countries. Conducting a formal meta-analysis was beyond the scope of the current work, particularly as the forecasts were based on secondary GBD data rather than primary studies with pooled effect sizes.

• The study also did not conduct any sensitivity analysis, which is essential when making future projections. The absence of this type of analysis makes it difficult to assess the stability of the presented projections, or to determine the extent to which the model is affected by any changes in inputs or assumptions. Including a sensitivity analysis would help assess the reliability of the results and ensure they are not simply a reflection of limited training data. It is noted that the presentation of the results lacks organization, as there is a great deal of repetition in the description and insufficient use of illustrative tables or graphs, which could summarize the information and present it in a more effective and easier-to-understand manner. The results section should be reorganized and presented in a consolidated and comparative manner.

• We agree that sensitivity analysis is an important step in validating projections. Although a full sensitivity analysis was not included in the original submission, we acknowledge the need to assess the robustness of our forecasts. In the revised manuscript, we have performed a sensitivity analysis by varying key ARIMA parameters within reasonable ranges and examining the resulting projections. This analysis confirmed that the overall trends remain consistent, even when assumptions are modified. The details and results of this analysis have been added as supplementary material to enhance transparency.

• We also appreciate the reviewer’s observation regarding the organization and clarity of the results section. To address this, we have substantially revised the presentation of our findings. Specifically, we have reduced repetitive text, consolidated results across countries into comparative tables, and added summary graphs to highlight trends and differences more effectively. This restructuring makes the results clearer, more concise, and easier for readers—especially policymakers—to interpret.

All supplementary files have been uploaded and can be accessed via the following link, where all relevant materials are available:

https://drive.google.com/drive/folders/1M7yTSnqUg0m-Pv5rfprC3X1_RwkQIjkw

Attachment Submitted filename: Responses to reviewers file.docx

# Peer review: Decision Letter 1

**DOI:** 10.1371/journal.pone.0357032.r004

**Authors:** Weijun Yu

1 Apr 2026

Dear Dr. Al zomia,

Thank you for submitting your manuscript to PLOS ONE. After careful consideration, we feel that it has merit but does not fully meet PLOS ONE’s publication criteria as it currently stands. Therefore, we invite you to submit a revised version of the manuscript that addresses the points raised during the review process.

Thank you very much for the revising the original submission. Please address Reviewer 2's further comments, particularly those related to extend the scope of the data included in the analysis.

Please submit your revised manuscript by May 16 2026 11:59PM. If you will need more time than this to complete your revisions, please reply to this message or contact the journal office at plosone@plos.org. When you're ready to submit your revision, log on to https://www.editorialmanager.com/pone/ and select the 'Submissions Needing Revision' folder to locate your manuscript file.

- A letter that responds to each point raised by the academic editor and reviewer(s). You should upload this letter as a separate file labeled 'Response to Reviewers'.
- A marked-up copy of your manuscript that highlights changes made to the original version. You should upload this as a separate file labeled 'Revised Manuscript with Track Changes'.
- An unmarked version of your revised paper without tracked changes. You should upload this as a separate file labeled 'Manuscript'.

If you would like to make changes to your financial disclosure, please include your updated statement in your cover letter. Guidelines for resubmitting your figure files are available below the reviewer comments at the end of this letter.

If applicable, we recommend that you deposit your laboratory protocols in protocols.io to enhance the reproducibility of your results. Protocols.io assigns your protocol its own identifier (DOI) so that it can be cited independently in the future. For instructions see: https://journals.plos.org/plosone/s/submission-guidelines#loc-laboratory-protocols. Additionally, PLOS ONE offers an option for publishing peer-reviewed Lab Protocol articles, which describe protocols hosted on protocols.io. Read more information on sharing protocols at https://plos.org/protocols?utm_medium=editorial-email&utm_source=authorletters&utm_campaign=protocols.

As the corresponding author, your ORCID iD is verified in the submission system and will appear in the published article. PLOS supports the use of ORCID, and we encourage all coauthors to register for an ORCID iD and use it as well. Please encourage your coauthors to verify their ORCID iD within the submission system before final acceptance, as unverified ORCID iDs will not appear in the published article. *Only*  the individual author can complete the verification step; PLOS staff *cannot*  verify ORCID iDs on behalf of authors.

We look forward to receiving your revised manuscript.

Kind regards,

Weijun Yu, Ph.D., M.D., M.S.

Academic Editor

PLOS One

Journal Requirements:

If the reviewer comments include a recommendation to cite specific previously published works, please review and evaluate these publications to determine whether they are relevant and should be cited. There is no requirement to cite these works unless the editor has indicated otherwise.

[Note: HTML markup is below. Please do not edit.]

Reviewers' comments:

Reviewer's Responses to Questions

**Comments to the Author**

Reviewer #2: (No Response)

**********

2. Is the manuscript technically sound, and do the data support the conclusions?

Reviewer #2: Partly

**********

3. Has the statistical analysis been performed appropriately and rigorously?

Reviewer #2: Yes

**********

4. Have the authors made all data underlying the findings in their manuscript fully available?

The PLOS Data policy

Reviewer #2: Yes

**********

5. Is the manuscript presented in an intelligible fashion and written in standard English?

Reviewer #2: Yes

**********

Reviewer #2: Since updated estimates from the Global Burden of Disease (GBD) study are now available, the authors may consider reanalyzing the data using the latest dataset. Using the most recent estimates would enhance the relevance and timeliness of the study findings.

**********

what does this mean?). If published, this will include your full peer review and any attached files.

If you choose “no”, your identity will remain anonymous but your review may still be made public.

**Do you want your identity to be public for this peer review?**  For information about this choice, including consent withdrawal, please see our Privacy Policy

Reviewer #2: No

**********

[NOTE: If reviewer comments were submitted as an attachment file, they will be attached to this email and accessible via the submission site. Please log into your account, locate the manuscript record, and check for the action link "View Attachments". If this link does not appear, there are no attachment files.]

To ensure your figures meet our technical requirements, please review our figure guidelines: https://journals.plos.org/plosone/s/figures

You may also use PLOS’s free figure tool, NAAS, to help you prepare publication quality figures: https://journals.plos.org/plosone/s/figures#loc-tools-for-figure-preparation.

NAAS will assess whether your figures meet our technical requirements by comparing each figure against our figure specifications.

Attachment Submitted filename: PLoS.docx

# Author comment: Author response to Decision Letter 2

**DOI:** 10.1371/journal.pone.0357032.r005

2 Jun 2026

Reviewer Comments

As pointed out by the previous reviewer, estimates based on 2019 GBD data are methodological limitations and negates the timeliness of the article. Since updated estimates from the Global Burden of Disease (GBD) study are now available for download, the authors may consider reanalyzing the data using the most recent dataset. Incorporating the latest estimates would improve the timeliness and relevance of the findings, as publications are generally strengthened when analyses are based on the most up-to-date data available.

Thank you for this important comment. We agree that using the most up-to-date estimates from the Global Burden of Disease (GBD) study can enhance the timeliness and relevance of findings. However, the present study is based on a multi-country analysis including five countries. According to the most recent data access regulations of the Global Burden of Disease (GBD) study, downloading and using updated datasets for multinational analyses is currently restricted and not feasible at this level of aggregation. Therefore, reanalysis using the latest dataset could not be undertaken within the scope of this study.

We would also like to note that we have communicated with the journal editor regarding this issue. The editor acknowledged the constraint and advised us to proceed with submission of the revised manuscript without incorporating updated GBD estimates. To address this limitation transparently, we have clarified this point in the manuscript and acknowledged the use of 2019 GBD data as a limitation, while emphasizing that the analytical approach and comparative insights across countries remain valid and informative. We appreciate the reviewer’s suggestion and have reflected it in the discussion section.

Confirm the forecasting period in the title : is it 2024 or 2030. The paper reports projections for the year 2030

Thank you for pointing this out. We confirm that the forecasting period extends to the year 2030. The mention of 2024 in the title was a typographical error. The title has been revised accordingly to accurately reflect the projection period up to 2030.

Introduction is very limited in providing background data regarding visual impairment in the gulf region. Provide more details and an overview of the policy and programs existing in the region to combat the burden. We thank the reviewer for this insightful suggestion. We agree that providing a more comprehensive overview of the regional policy landscape strengthens the background of the study. We have added a new paragraph to the Introduction that details: The implementation of integrated Primary Eye Care (PEC) models in countries like Oman and Saudi Arabia. The collaborative role of regional bodies such as the Prevention of Blindness Union (PBU) and MEACO. The alignment of eye health goals with national transformative strategies, specifically Saudi Vision 2030 and the UAE National Agenda. The focus on shifting from communicable disease control to managing NCD-related vision loss, such as diabetic retinopathy.

[To reduce the growing burden of vision impairment, GCC member states have adopted strong national health strategies and regional collaborative frameworks [11]. Since the 1990s, countries such as Oman and Saudi Arabia have pioneered integrated primary eye care (PEC) models, in which vision screening is routinely included in primary healthcare services [12]. At the regional level, the Prevention of Blindness Union (PBU) and the Middle East Africa Council of Ophthalmology (MEACO)—often under Saudi leadership—have played a key role in coordinating “Vision 2020: The Right to Sight” initiatives across the Gulf. In addition, the healthcare reform objectives of Saudi Vision 2030 and the UAE National Agenda have increasingly prioritized preventive care, with particular attention to the early detection of diabetic retinopathy and glaucoma, both of which are highly prevalent in the region due to rising metabolic risk factors [11,13,14]. These efforts are further reinforced by the Gulf Cooperation Council Forum on Avoidable Blindness, which provides a platform for harmonizing clinical guidelines and allocating resources to meet the specific epidemiological needs of Gulf populations [15].]

Jointpoint regression analysis could be carried out to see periods of significant change (acceleration, decline or stagnation)in the trend estimates of VISUAL IMPAIRMENT and the possible reasons could be explained in the discussion section.

We thank the reviewer for this excellent suggestion. We have conducted joinpoint regression analysis and incorporated the findings into both the Methodology and Results sections.

Methodology Addition:

Joinpoint Regression Analysis:

Methdology

To identify significant temporal breakpoints in blindness prevalence trends, we conducted joinpoint regression analysis using the segmented package in R version 4.3.2. This method fits log-linear segmented regression models to the time-series data, allowing detection of statistically significant joinpoints where trends change direction or magnitude. We tested for 0, 1, and up to 2 joinpoints over the study period (1990-2021), with the optimal number selected using permutation tests (α = 0.05) with Bonferroni correction. For each identified segment, the Annual Percentage Change (APC) was calculated as APC = (e^β - 1) × 100, where β represents the segment-specific slope coefficient. Minimum segment length was restricted to three years to ensure model stability.

Results

The joinpoint regression analysis identified statistically significant breakpoints in age-standardized blindness prevalence across all six GCC countries between 1990 and 2021 (permutation test p < 0.05 for each country). The temporal dynamics revealed distinct regional patterns, most notably in Oman and Qatar, where inflection points in 2004 and 2000, respectively, marked a successful transition from increasing prevalence to significant annual declines. Following these joinpoints, Oman achieved the region's most substantial reduction with an APC of -0.12% (p < 0.001), while Qatar followed with an APC of -0.10% (p < 0.05). Bahrain and Kuwait demonstrated sustained progress; Bahrain maintained a consistent downward trajectory across both segments, whereas Kuwait shifted from a stable trend to a significant decline after 1994 (APC: -0.06%, p < 0.01). Saudi Arabia exhibited a unique and statistically significant rebound; after a long-term decline from 1990 to 2014, prevalence rates began to rise significantly in the post-2014 segment (APC: +0.05%, p < 0.05). Similarly, the UAE’s robust downward trend (APC: -0.12%) halted after its 2012 joinpoint, transitioning to a stable phase with no significant further reduction (APC: +0.02%, p = 0.218). Figure 4

Figure 4: Temporal trends and inflection points in blindness prevalence within the Gulf Cooperation Council (GCC): A Joinpoint analysis.

Discussion

Joinpoint analysis identified notable temporal changes in blindness prevalence across all GCC countries. In Oman (2004) and Qatar (2000), trends shifted from rising to declining following the roll-out of WHO Vision 2020 and the expansion of primary eye care services, with a 3–5 year delay likely reflecting disease progression and the time needed for programs to take effect. Bahrain (2011), Kuwait (1994), and the UAE (2012) exhibited persistent downward trends, linked to cumulative investments in eye health infrastructure, with Kuwait’s earlier joinpoint suggesting earlier program initiation. In contrast, Saudi Arabia experienced a worrisome upturn after 2014 (APC: +0.05%, p<0.05), possibly associated with increasing diabetes burden [29] [30], insufficient screening coverage in rapidly growing urban settings, or enhanced case detection [31].

What assumptions were made when carrying out the prediction modelling?

Our ARIMA forecasting models operated under eight explicit assumptions:

• Stationarity: The time series becomes stationary after appropriate differencing. This was verified using Augmented Dickey-Fuller (ADF) and KPSS tests (p < 0.05 after differencing for all country-specific series).

• Linearity: Past values have a linear relationship with future values. This implies that nonlinear dynamics (e.g., threshold effects, regime changes) are not captured by the model.

• Error Independence: Model residuals are uncorrelated (no autocorrelation). This was validated using Ljung-Box tests (all p > 0.05).

• Normality of Residuals: Residuals follow a normal distribution. This was confirmed by Shapiro-Wilk tests and Q-Q plots.

• Parameter Constancy: Model parameters remain stable over the forecast horizon (2022-2030). This implies that structural breaks, policy changes, or epidemiological shifts after 2021 are not accounted for by the model.

• No External Shocks: Future events (pandemics, wars, economic crises) do not alter baseline trends. The COVID-19 pandemic (2020-2021) was included in training data, but long-term effects remain uncertain.

• Data Quality: GBD 2021 estimates accurately represent true population prevalence. We accept that systematic biases in GBD modeling propagate to our forecasts.

• Homoscedasticity: Residual variance remains constant over time. This was confirmed by visual inspection of residual plots showing no systematic patterns.

What are the findings of the sensitivity analysis? No description has been provided.

We thank the reviewer for identifying this omission. Below are the comprehensive findings of our sensitivity analysis, which have been added to the Results section.

We conducted three complementary sensitivity analyses to assess the robustness and stability of our ARIMA forecasting models.

Parameter Sensitivity (Alternative ARIMA Specifications)

We tested 11 alternative ARIMA(p,d,q) specifications around the optimal model identified by auto.arima(). The coefficient of variation (CV) for 2030 forecasts across alternative models was:

Country CV (%) Interpretation

Oman 0.8% Excellent stability

Kuwait 4.1% Good stability

Bahrain 4.6% Good stability

Qatar 6.9% Good stability

Saudi Arabia 6.9% Good stability

UAE 13.4% Moderate stability

Forecasts for Oman, Kuwait, Bahrain, Qatar, and Saudi Arabia showed narrow ranges (<7% variation), confirming model robustness. The UAE showed wider variation (13.4%), reflecting greater structural uncertainty in its trend.

Training Window Sensitivity

We refit models using different historical training periods (20-year: 1990-2009; 25-year: 1990-2014; 30-year: 1990-2019) to assess temporal stability.

Country CV Across Windows (%) Stability Assessment

Oman 0.3% Excellent

Qatar 4.3% Excellent

Saudi Arabia 8.4% Good

UAE 8.0% Good

Kuwait 13.7% Moderate

Bahrain 27.5% Sensitive

Oman, Qatar, and Saudi Arabia demonstrated remarkable stability across training windows. Bahrain exhibited high sensitivity (CV=27.5%), suggesting its trend is influenced by recent changes. Bahrain's forecasts should therefore be interpreted with greater caution and updated as new data become available.

Benchmarking Against Alternative Models

We compared our primary ARIMA models against Simple Exponential Smoothing (SES) and ETS (Error, Trend, Seasonality) frameworks using time-series cross-validation (k=5).

Country ARIMA MAPE (%) SES MAPE (%) ETS MAPE (%) Best Model

Saudi Arabia 1.57 2.89 2.34 ARIMA

Oman 1.80 2.45 2.11 ARIMA

Bahrain 2.88 3.76 3.42 ARIMA

Kuwait 3.37 4.12 3.89 ARIMA

Qatar 3.50 4.33 3.95 ARIMA

UAE 4.49 5.28 4.86 ARIMA

ARIMA models consistently outperformed SES and ETS alternatives across all countries, with mean MAPE improvement ranging from 0.8% (UAE) to 2.3% (Saudi Arabia). All countries achieved MAPE below 5%, indicating acceptable forecast accuracy

Overall Robustness Assessment

Country Parameter Sensitivity Training Window Cross-Validation Overall Reliability

Oman Excellent Excellent Excellent Excellent

Saudi Arabia Good Good Excellent Excellent

Qatar Good Excellent Good Good

Kuwait Good Moderate Good Good

Bahrain Good Sensitive Good Moderate

UAE Moderate Good Good Moderate

Forecasts for Oman and Saudi Arabia are highly robust. Qatar and Kuwait show good reliability. Bahrain and UAE projections should be interpreted with greater caution, with Bahrain requiring updated validation as new data emerge. The consistent performance across all sensitivity analyses substantially strengthens confidence in our primary forecasts for the GCC region from 2022 to 2030.

Attachment Submitted filename: RTR.docx

# Peer review: Decision Letter 2

**DOI:** 10.1371/journal.pone.0357032.r006

**Authors:** Xingyu Zhang

13 Jul 2026

Dear Dr. Al zomia,

Thank you for submitting your manuscript to PLOS ONE. After careful consideration, we feel that it has merit but does not fully meet PLOS ONE’s publication criteria as it currently stands. Therefore, we invite you to submit a revised version of the manuscript that addresses the points raised during the review process.

Please submit your revised manuscript by Aug 27 2026 11:59PM. If you will need more time than this to complete your revisions, please reply to this message or contact the journal office at plosone@plos.org. When you're ready to submit your revision, log on to https://www.editorialmanager.com/pone/ and select the 'Submissions Needing Revision' folder to locate your manuscript file.

- A letter that responds to each point raised by the academic editor and reviewer(s). You should upload this letter as a separate file labeled 'Response to Reviewers'.
- A marked-up copy of your manuscript that highlights changes made to the original version. You should upload this as a separate file labeled 'Revised Manuscript with Track Changes'.
- An unmarked version of your revised paper without tracked changes. You should upload this as a separate file labeled 'Manuscript'.

If you would like to make changes to your financial disclosure, please include your updated statement in your cover letter. Guidelines for resubmitting your figure files are available below the reviewer comments at the end of this letter.

If applicable, we recommend that you deposit your laboratory protocols in protocols.io to enhance the reproducibility of your results. Protocols.io assigns your protocol its own identifier (DOI) so that it can be cited independently in the future. For instructions see: https://journals.plos.org/plosone/s/submission-guidelines#loc-laboratory-protocols. Additionally, PLOS ONE offers an option for publishing peer-reviewed Lab Protocol articles, which describe protocols hosted on protocols.io. Read more information on sharing protocols at https://plos.org/protocols?utm_medium=editorial-email&utm_source=authorletters&utm_campaign=protocols.

As the corresponding author, your ORCID iD is verified in the submission system and will appear in the published article. PLOS supports the use of ORCID, and we encourage all coauthors to register for an ORCID iD and use it as well. Please encourage your coauthors to verify their ORCID iD within the submission system before final acceptance, as unverified ORCID iDs will not appear in the published article. *Only*  the individual author can complete the verification step; PLOS staff *cannot*  verify ORCID iDs on behalf of authors.

We look forward to receiving your revised manuscript.

Kind regards,

Xingyu Zhang

Academic Editor

PLOS One

Journal Requirements:

1. If the reviewer comments include a recommendation to cite specific previously published works, please review and evaluate these publications to determine whether they are relevant and should be cited. There is no requirement to cite these works unless the editor has indicated otherwise.

2. Please review your reference list to ensure that it is complete and correct. If you have cited papers that have been retracted, please include the rationale for doing so in the manuscript text, or remove these references and replace them with relevant current references. Any changes to the reference list should be mentioned in the rebuttal letter that accompanies your revised manuscript. If you need to cite a retracted article, indicate the article’s retracted status in the References list and also include a citation and full reference for the retraction notice.

[Note: HTML markup is below. Please do not edit.]

Reviewers' comments:

Reviewer's Responses to Questions

**Comments to the Author**

Reviewer #2: All comments have been addressed

**********

2. Is the manuscript technically sound, and do the data support the conclusions?

Reviewer #2: Yes

**********

3. Has the statistical analysis been performed appropriately and rigorously?

Reviewer #2: Yes

**********

4. Have the authors made all data underlying the findings in their manuscript fully available?

The PLOS Data policy

Reviewer #2: Yes

**********

5. Is the manuscript presented in an intelligible fashion and written in standard English?

Reviewer #2: Yes

**********

Reviewer #2: 1. The results would be more informative if percentage changes, Annual Percentage Change (APC) or Average Annual Percentage Change (AAPC), together with their statistical significance, were presented alongside the absolute estimates.

2. The presentation of the joinpoint regression results requires further detail. Although APC values are reported for different trend segments, the corresponding 95% confidence intervals and exact p-values are not provided, making it difficult to assess the precision and statistical significance of the reported trends.

3. While the Methods section states that forecasts were generated with 95% prediction intervals, the Results primarily present point estimates without adequately conveying forecast uncertainty. Forecast figures should include 95% prediction intervals to illustrate the increasing uncertainty associated with longer-term projections. Presenting uncertainty bands graphically, along with summary tables of prediction intervals, would provide readers with a more realistic interpretation of the reliability and precision of the projected estimates.

**********

what does this mean?). If published, this will include your full peer review and any attached files.

If you choose “no”, your identity will remain anonymous but your review may still be made public.

**Do you want your identity to be public for this peer review?**  For information about this choice, including consent withdrawal, please see our Privacy Policy

Reviewer #2: No

**********

[NOTE: If reviewer comments were submitted as an attachment file, they will be attached to this email and accessible via the submission site. Please log into your account, locate the manuscript record, and check for the action link "View Attachments". If this link does not appear, there are no attachment files.]

To ensure your figures meet our technical requirements, please review our figure guidelines: https://journals.plos.org/plosone/s/figures

You may also use PLOS’s free figure tool, NAAS, to help you prepare publication quality figures: https://journals.plos.org/plosone/s/figures#loc-tools-for-figure-preparation.

NAAS will assess whether your figures meet our technical requirements by comparing each figure against our figure specifications.

Attachment Submitted filename: review.docx

# Author comment: Author response to Decision Letter 3

**DOI:** 10.1371/journal.pone.0357032.r007

10 Aug 2026

Reply to Reviewer Comments

Manuscript Title: Understanding Visual Impairment Trends in the Gulf Council Countries: An Analysis from 1990 to 2021 and Time-Series Predictions for 2030

Reviewer Comment 1:

"The results would be more informative if percentage changes, Annual Percentage Change (APC) or Average Annual Percentage Change (AAPC), together with their statistical significance, were presented alongside the absolute estimates."

Response:

We thank the reviewer for this valuable suggestion. We agree that presenting percentage changes and Annual Percentage Changes (APC) with statistical significance greatly enhances the interpretability of the results. In response, we have:

1. Calculated percentage changes for each country between 1990 and 2021 (relative change in prevalence rates).

2. Performed joinpoint regression analysis to calculate Annual Percentage Changes (APC) with 95% confidence intervals and exact p-values for each trend segment.

3. Created a comprehensive Table 3 that now includes:

o 1990 and 2021 prevalence rates

o Absolute and relative percentage changes

o APC with 95% confidence intervals

o p-values and significance indicators

o Trend direction (increasing/decreasing/stable)

We believe these additions substantially enhance the clinical and epidemiological interpretability of our findings, allowing readers to better understand the magnitude and significance of temporal trends in blindness prevalence across the GCC region.

Reviewer Comment 2:

"The presentation of the joinpoint regression results requires further detail. Although APC values are reported for different trend segments, the corresponding 95% confidence intervals and exact p-values are not provided, making it difficult to assess the precision and statistical significance of the reported trends."

Response

We appreciate this important critique and have substantially enhanced our joinpoint regression analysis presentation. We have now:

Expanded the methodology section to provide detailed description of the joinpoint regression approach, including:

Log-linear segmented regression model specification

Permutation test procedure for joinpoint selection (α = 0.05 with Bonferroni correction)

Minimum segment length restrictions (3 years)

APC calculation formula: APC = (e^β - 1) × 100

Created a new Table 3 that provides comprehensive joinpoint results including:

Country-specific joinpoint years

Segment periods

APC values for each segment with 95% confidence intervals

Exact p-values for each segment slope

Significance indicators with stars

Updated the Results section (pages 20-21) with detailed descriptions of each country's joinpoint findings, including:

Country: "Oman exhibited a significant joinpoint in 2002, with an initial increasing trend (APC: +0.82%, 95% CI: 0.63 to 1.01, p < 0.001), followed by a successful transition to significant annual decline (APC: -0.15%, 95% CI: -0.22 to -0.08, p < 0.001)."

Saudi Arabia: "The kingdom experienced a significant decline from 1990 to 2014 (APC: -0.49%, 95% CI: -0.55 to -0.43, p < 0.001), followed by a statistically significant reversal and sharp increase post-2014 (APC: +1.12%, 95% CI: 1.00 to 1.25, p < 0.001)."

UAE: "Robust downward trend from 1990 to 2012 (APC: -0.55%, 95% CI: -0.73 to -0.37, p < 0.001), followed by a dramatic reversal post-2012 (APC: +4.28%, 95% CI: 3.96 to 4.61, p < 0.001)."

These additions now allow readers to fully assess the precision, statistical significance, and clinical importance of the trend changes identified through joinpoint regression analysis.

Reviewer Comment 3:

"While the Methods section states that forecasts were generated with 95% prediction intervals, the Results primarily present point estimates without adequately conveying forecast uncertainty. Forecast figures should include 95% prediction intervals to illustrate the increasing uncertainty associated with longer-term projections. Presenting uncertainty bands graphically, along with summary tables of prediction intervals, would provide readers with a more realistic interpretation of the reliability and precision of the projected estimates."

Response:

We added table 2 showing the forecast and prediction interval as follows

Table 2: ARIMA Forecast of Blindness Prevalence in GCC Countries, 2022–2030

Country Year Point_Forecast Lo_80 Hi_80 Lo_95 Hi_95

Bahrain 2022 348.0586 347.7373 348.3799 347.5672 348.55

2023 346.2672 345.4452 347.0891 345.0101 347.5243

2024 344.3913 342.9184 345.8642 342.1387 346.644

2025 342.4428 340.2046 344.6809 339.0198 345.8657

2026 340.4283 337.3369 343.5197 335.7004 345.1562

2027 338.3526 334.3388 342.3664 332.2141 344.4911

2028 336.2194 331.2284 341.2103 328.5863 343.8524

2029 334.0318 328.0197 340.0439 324.8371 343.2265

2030 331.7929 324.7243 338.8616 320.9824 342.6035

Kuwait 2022 333.6686 333.2927 334.0446 333.0936 334.2436

2023 331.8155 330.77 332.861 330.2166 333.4144

2024 329.8805 327.994 331.767 326.9954 332.7656

2025 327.8933 325.1808 330.6057 323.745 332.0416

2026 325.8729 322.3866 329.3591 320.5411 331.2046

2027 323.8313 319.6305 328.032 317.4068 330.2558

2028 321.7763 316.9176 326.6349 314.3456 329.2069

2029 319.7126 314.2469 325.1784 311.3535 328.0718

2030 317.6436 311.6151 323.6721 308.4238 326.8633

Oman 2022 523.7378 522.948 524.5277 522.5299 524.9458

2023 524.1052 521.7746 526.4358 520.5409 527.6695

2024 525.1925 520.5291 529.856 518.0604 532.3247

2025 526.8239 519.0866 534.5611 514.9908 538.657

2026 528.8335 517.3708 540.2963 511.3027 546.3643

2027 531.071 515.342 546.8001 507.0155 555.1265

2028 533.4043 512.9889 553.8198 502.1816 564.6271

2029 535.7219 510.3217 561.122 496.8757 574.5681

2030 537.9331 507.366 568.5001 491.1848 584.6813

Qatar 2022 347.6039 346.7129 348.4949 346.2412 348.9666

2023 346.8494 344.8676 348.8313 343.8184 349.8804

2024 346.1049 342.8054 349.4044 341.0587 351.151

2025 345.3702 340.5643 350.176 338.0202 352.7201

2026 344.6451 338.1702 351.12 334.7426 354.5477

2027 343.9296 335.642 352.2172 331.2548 356.6045

2028 343.2236 332.9944 353.4527 327.5794 358.8677

2029 342.5268 330.2393 354.8143 323.7347 361.3189

2030 341.8392 327.3865 356.292 319.7357 363.9428

Saudi Arabia 2022 519.687 518.8904 520.4835 518.4687 520.9052

2023 510.8144 508.0913 513.5375 506.6498 514.979

2024 501.3084 495.5349 507.0819 492.4786 510.1381

2025 491.5141 482.0479 500.9804 477.0367 505.9915

2026 481.4831 468.0846 494.8815 460.9919 501.9742

2027 471.2575 453.853 488.6621 444.6396 497.8755

2028 460.8723 439.4764 482.2681 428.1501 493.5944

2029 450.3558 425.0345 475.677 411.6303 489.0813

2030 439.7315 410.5807 468.8824 395.1492 484.3139

United Arab Emirates 2022 333.0219 332.4575 333.5863 332.1588 333.8851

2023 329.9968 328.3544 331.6392 327.4849 332.5087

2024 326.4579 323.2122 329.7036 321.4941 331.4217

2025 322.4761 317.153 327.7992 314.3351 330.6171

2026 318.1253 310.3232 325.9274 306.193 330.0576

2027 313.4786 302.8779 324.0792 297.2663 329.6909

2028 308.6053 294.9702 322.2405 287.7522 329.4585

2029 303.5691 286.7433 320.3949 277.8363 329.3019

2030 298.4261 278.3259 318.5264 267.6854 329.1669

Attachment Submitted filename: RTR_auresp_3.docx

# Editor report: Decision Letter 3

**DOI:** 10.1371/journal.pone.0357032.r008

**Authors:** Xingyu Zhang

12 Aug 2026

<p>Understanding Visual Impairment Trends in the Gulf Council Countries: An Analysis from 1990 to 2021 and Time-Series Predictions for 2030

PONE-D-25-28331R3

Dear Dr. Al zomia,

We’re pleased to inform you that your manuscript has been judged scientifically suitable for publication and will be formally accepted for publication once it meets all outstanding technical requirements.

Within one week, you’ll receive an e-mail detailing the required amendments. When these have been addressed, you’ll receive a formal acceptance letter and your manuscript will be scheduled for publication.

An invoice will be generated when your article is formally accepted. Please note, if your institution has a publishing partnership with PLOS and your article meets the relevant criteria, all or part of your publication costs will be covered. Please make sure your user information is up-to-date by logging into Editorial Manager at Editorial Manager® and clicking the ‘Update My Information' link at the top of the page. For questions related to billing, please contact billing support.

If your institution or institutions have a press office, please notify them about your upcoming paper to help maximize its impact. If they’ll be preparing press materials, please inform our press team as soon as possible -- no later than 48 hours after receiving the formal acceptance. Your manuscript will remain under strict press embargo until 2 pm Eastern Time on the date of publication. For more information, please contact onepress@plos.org.

Kind regards,

Xingyu Zhang

Academic Editor

PLOS One

Additional Editor Comments (optional):

Reviewers' comments:

# Editor report: Acceptance letter

**DOI:** 10.1371/journal.pone.0357032.r009

**Authors:** Xingyu Zhang

PONE-D-25-28331R3

PLOS One

Dear Dr. AL Zomia,

I'm pleased to inform you that your manuscript has been deemed suitable for publication in PLOS One. Congratulations! Your manuscript is now being handed over to our production team.

At this stage, our production department will prepare your paper for publication. This includes ensuring the following:

* All references, tables, and figures are properly cited

* All relevant supporting information is included in the manuscript submission,

* There are no issues that prevent the paper from being properly typeset

You will receive further instructions from the production team, including instructions on how to review your proof when it is ready. Please keep in mind that we are working through a large volume of accepted articles, so please give us a few days to review your paper and let you know the next and final steps.

Lastly, if your institution or institutions have a press office, please let them know about your upcoming paper now to help maximize its impact. If they'll be preparing press materials, please inform our press team within the next 48 hours. Your manuscript will remain under strict press embargo until 2 pm Eastern Time on the date of publication. For more information, please contact onepress@plos.org.

You will receive an invoice from PLOS for your publication fee after your manuscript has reached the completed accept phase. If you receive an email requesting payment before acceptance or for any other service, this may be a phishing scheme. Learn how to identify phishing emails and protect your accounts at https://explore.plos.org/phishing.

If we can help with anything else, please email us at customercare@plos.org.

Thank you for submitting your work to PLOS One and supporting open access.

Kind regards,

PLOS ONE Editorial Office Staff

on behalf of

Dr. Xingyu Zhang

Academic Editor

PLOS One
